# Adaptive Evolution Reveals Metabolic Plasticity and Functional Redundancy in an Anaerobic Microbiome under Extreme Ammonia Stress

**DOI:** 10.1021/acs.est.6c05759

**Published:** 2026-08-26

**Authors:** Luca Francescato, Gabriele Ghiotto, Maria Chiara Valerin, Nicola De Bernardini, Sofia Fraulini, Annalisa Sandon, Laura Treu, Maria Cristina Lavagnolo, Stefano Campanaro

**Affiliations:** † Department of Biology, 124236 University of Padova, Via U. Bassi 58/b, Padova 35121, Italy; ‡ Joint Genome Institute, Lawrence Berkeley National Laboratory, 1 Cyclotron Road, Berkeley, California 94720, United States; § Department of Civil, Environmental and Architectural Engineering, 9308 University of Padova, Via Marzolo 9, Padova 35131, Italy

**Keywords:** Anaerobic digestion, Ammonia inhibition, Metagenomics, Metatranscriptomics, Osmoprotectant

## Abstract

Ammonia toxicity represents a primary biochemical bottleneck governing microbial community structure and performance during the anaerobic digestion of the organic fraction of municipal solid waste. However, the mechanistic basis of microbial adaptation to chronic ammonia levels remains poorly characterized. In this study, a long-term sequential enrichment strategy under progressively increasing ammonia concentrations (350–1500 mgN L^–1^), integrated with genome-centric metagenomics and metatranscriptomics, was employed to resolve the response of an organic waste-degrading microbiome over a 240 day period. Increasing ammonia pressure induced a progressive decline in methanogenesis and accumulation of volatile fatty acids, particularly acetate. Despite these inhibitory pressures, methane production was only halved relative to the initial baseline reflecting a resilient methanogenic community. This stability was driven by a restructuring of the microbiome, where functional redundancy across divergent taxa preserved core metabolic functions. Key adaptive responses included the reconfiguration of carbon fixation pathways, specifically via a variant of the Wood–Ljungdahl pathway coupled with the glycine cleavage system acting as an alternative acetate oxidation route, as well as sustained osmoprotectant biosynthesis. Cellular homeostasis was preserved through H^+^ replenishment via multiple energy-converting complexes and K^+^ influx to maintain cation–proton balance. Collectively, these findings demonstrate that metabolic plasticity and the preservation of core metabolic functions are the primary determinants of ammonia resilience, sustaining methane production under inhibitory conditions.

## Graphical abstract

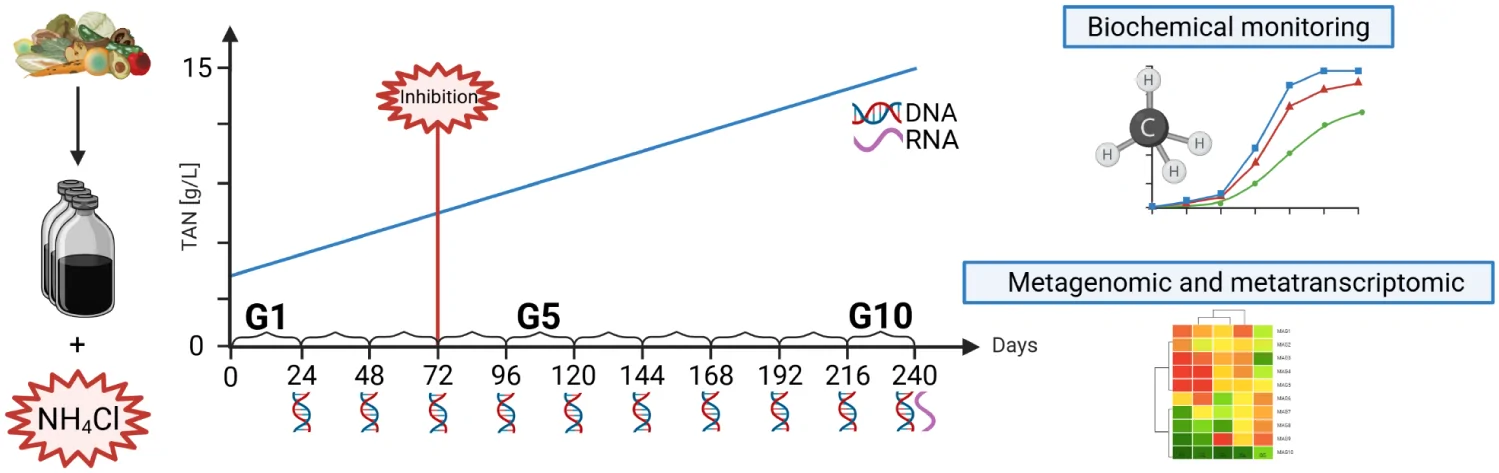

## 1 Introduction

Global municipal solid waste generation is estimated at around 2 billion tonnes per year and is expected to increase further, posing major challenges to environmental sustainability and public health.^1,2^ The organic fraction of municipal solid waste (OFMSW) accounts for 42–69% of the total and represents a key stream requiring effective management.^2^ Its composition is highly heterogeneous, varying with seasonal trends, collection systems, and socio-economic conditions.^3,4^ OFMSW mainly consists of food waste, along with garden residues, paper, and other cellulosic materials.^5–7^ It includes lipids, proteins, and carbohydrates,^6,8^ as well as structural polymers such as lignin. This compositional variability strongly influences the efficiency and stability of the treatment and valorization processes. Despite increasing regulatory pressure to reduce landfill disposal and promote sustainable waste management, a significant fraction of OFMSW is still disposed of through landfilling or incineration, leading to greenhouse gas emissions and leachate-related soil and groundwater contamination.^9,10^

Among alternative treatments, anaerobic digestion (AD) stands out as a well-established biotechnological process converting organic matter into renewable energy and value-added products, primarily in the form of biogas and digestate.^10–12^ AD proceeds through four sequential stages: hydrolysis, acidogenesis, acetogenesis, and methanogenesis.^13^ The latter is responsible for methane (CH_4_) production but is also the most sensitive to operational stressors, including pH imbalances, heavy metals, and elevated ammonia (NH_3_) loads.^14^ NH_3_ inhibition is critical when treating nitrogen-rich substrates, where protein degradation leads to the release of reactive nitrogen species.^15^ In aqueous systems, total ammonia nitrogen (TAN) exists in equilibrium between ionized ammonium (NH_4_^+^) and un-ionized free ammonia nitrogen (NH_3_, FAN), whose relative distribution depends strictly on pH and temperature.^15^ While moderate NH_3_ levels can support microbial growth, excessive FAN is toxic. It diffuses passively across cell membranes, disrupts intracellular pH, and inhibits enzymatic activity, impairing methanogenesis. Microorganisms respond by trying to maintain homeostasis, exporting ions (K^+^, Na^+^) at the cost of ATP.^2,16^ Additionally, osmolytes such as glutamate, glutamine, glycine-betaine, and Nε-acetyl-l-lysine are produced to regulate ion balance, mitigate osmotic stress, and stabilize proteins by preventing denaturation or misfolding.^17–20^ The relative contribution of these homeostatic strategies, however, remains poorly resolved at the community level, and whether they are sustained through de novo biosynthesis or the energetically cheaper uptake of compatible solutes is a question directly addressed by the present study.

Reported inhibitory thresholds for NH_3_ vary widely, from 27 to 1450 mg FAN L^–1^.^21^ This broad range stems largely from the strong dependence of FAN on pH and temperature, so that comparable TAN concentrations can correspond to very different FAN levels across studies. It is further amplified by a range of characteristics including differences in inoculum composition and prior acclimation history, substrate composition and operating mode, all of which shape the sensitivity and adaptive capacity of the community.^15,21^ Among the core AD microbiota, methanogenic archaea are the most sensitive,^22,23^ especially acetoclastic methanogens.^24–26^ Under high NH_3_ conditions, the acetoclastic route is often suppressed, favoring syntrophic acetate oxidation (SAO),^27^ where acetate is converted into carbon dioxide (CO_2_) and hydrogen (H_2_), which subsequently fuel hydrogenotrophic methanogens. While this transition often triggers process imbalances, volatile fatty acid (VFA) accumulation and reduced CH_4_ yields, the resulting pH drop can mitigate NH_3_ inhibition, leading to an unstable “inhibited steady state”.^28^ Despite extensive knowledge of these process-level consequences of NH_3_ inhibition, ecological and metabolic mechanisms that enable microbial communities to maintain functionality under prolonged NH_3_ stress remain only partially understood. Previous studies have identified NH_3_-tolerant taxa, osmoadaptation strategies, and shifts toward SAO, yet the links between community turnover, metabolic reorganization, and transcriptional activity remain poorly resolved. In particular, it is still unclear how changes in microbial composition translate into functional adaptation and which metabolic pathways are actively maintained within NH_3_-adapted communities.

In this study, a long-term enrichment strategy was employed over eight months to select a microbial community resilient to increasing FAN levels via stepwise addition of NH_4_Cl. A genome-centric metagenomic and metatranscriptomic approach was applied to investigate community dynamics, stability, and activity. Metabolic countermeasures driving resilience were identified in the key microbiome species. The results provide insights into the metabolic strategies enabling resilience, contributing to the development of robust microbial consortia for stable and efficient biogas production under inhibitory conditions.

## 2 Materials and Methods

### 2.1 Feedstock Characterization and Processing

The OFMSW, serving as the primary carbon source for the enrichment experiment, was derived from a waste treatment plant located in Northern Italy. The raw substrate was chemically characterized prior to processing, showing a pH of 3.7, a total solids (TS) content of 69.4 g kg^–1^ (86% of which volatile solids, VS), a total organic carbon (TOC) concentration of 31.0 gC kg^–1^, total Kjeldahl nitrogen (TKN) of 2.87 gN kg^–1^, total ammonia nitrogen (TAN) of 0.33 gN kg^–1^, and total phosphorus of 0.25 gP kg^–1^. To obtain a clarified and standardized feedstock, the solid fraction was removed by centrifugation at 10,000 *g* for 20 min (Avanti J-25, Beckman Coulter, USA), followed by supernatant filtration through a 3 μm pore-size paper filter (Millipore, Germany). The clarified liquid was autoclaved at 121 °C for 20 min to eliminate indigenous microbial populations. After processing, the substrate showed a reduced solids content (TS = 38.0 g L^–1^, VS = 78% of TS) while maintaining comparable nutrient composition, with TOC of 18.3 gC L^–1^, TKN of 1.26 gN L^–1^, TAN of 0.45 gN L^–1^, and total phosphorus of 0.24 gP L^–1^.

### 2.2 Inoculum and Experimental Setup

A microbial inoculum, supplied in lyophilized form by the industrial partner, was activated in Basal Anaerobic (BA) medium^29^ supplemented with 10 g L^–1^ Nutrient Broth (Merck, Germany) until CH_4_ production was detected. The enrichment experiment was conducted in triplicate in 120 mL serum bottles with a 50 mL working volume, using 10% (v/v) of the activated community as the initial inoculum. The growth medium consisted of OFMSW diluted in BA medium to reach 10 gC L^–1^ TOC, supplemented with 0.5 mL of yeast extract (20 g L^–1^; Merck, Germany) and 0.5 mL of Wolin’s vitamin solution to support initial microbial reactivation. The bottles were sealed with butyl rubber stoppers and aluminum crimp caps, flushed with N_2_ to establish anaerobic conditions, and incubated at 37 °C in an orbital shaker (NB-T205L, NBIOTEK, Korea) at 130 rpm. Preliminary assays were conducted to determine an appropriate NH_3_ baseline according to the recovery of methanogenic activity after inoculation, identifying 3.40 gN L^–1^ TAN (0.38 gN L^–1^ FAN) as the optimal starting concentration. A generation was defined as a 24-day period. This interval was chosen to accommodate eight stepwise additions of NH_4_Cl (0.6 g L^–1^ every 3 days), yielding a cumulative increment of 4 g L^–1^ NH_4_Cl (1 gN L^–1^ TAN) per generation while allowing the community sufficient time to metabolize the substrate and adapt to each new FAN level before the subsequent transfer. At the end of each generation, reinoculation was performed using 10% (v/v) of the previous culture, setting the TAN level accordingly. This procedure was adopted to establish successive enrichment cycles under progressively increasing NH_3_ concentrations, as demonstrated before.^18,30^ The selection of this NH_3_-tolerant community lasted 240 days with a total of 10 generations (G1–G10). All subsequent physicochemical measurements and performance metrics were gathered from each individual replicate, with data reported as the mean and error bars corresponding to standard deviations.

### 2.3 Culture Maintenance and Biochemical Analysis

Biogas production was monitored every 3 days. Gas volume was quantified by measuring headspace overpressure using a digital manometer (HD 2124.2, Delta Ohm, Italy), and its composition was determined by analyzing 5 mL headspace samples using a gas chromatograph (8860 GC, Agilent Technologies, Santa Clara, CA, USA) equipped with a thermal conductivity detector (TCD) and helium as the carrier gas. Sample processing for VFA quantification consisted first of the acidification to pH < 2 with orthophosphoric acid (85%, Sigma-Aldrich, Saint Louis, MO, USA), followed by centrifugation at 13,000 rpm for 10 min and filtration (0.22 μm). VFA were analyzed on days 3, 6, 9 and on the last day of each generation using an Agilent 8860 GC equipped with a flame ionization detector (FID) and a DB-FFAP fused-silica capillary column (30 m × 0.25 mm × 0.25 μm), with helium as the carrier gas. The pH was monitored every 3 days using a Basic 20 pH meter (Crison, Spain) and adjusted to 8.0 ± 0.1 through the addition of 1 M NaOH or HCl in order to maintain controlled FAN concentration. At the beginning and at the end of each generation, TAN was determined via steam distillation followed by titrimetric analysis with sulfuric acid, in accordance with the IRSA-CNR 29/2003 method (vol. 2, no. 4030C) to validate theoretical estimates. FAN values were calculated using the following equation:^31^

$$\mathrm{FAN}=\tan{\left(1+\frac{10^{-\mathrm{pH}}}{10^{-\left(0.09018+\frac{2729.92}{T\left(K\right)}\right)}}\right)}^{-1}$$

### 2.4 Nucleic Acid Extraction and Sequencing

For generations 1 to 5, genomic DNA was extracted from a total volume of 15 mL of culture, obtained by pooling together equal volume of each biological replicate, using the DNeasy PowerSoil Kit (Qiagen, Germany), with a modified protocol adding an initial phenol:chloroform:isoamyl alcohol step at a ratio of 25:24:1 (Sigma-Aldrich, Saint Louis, MO, USA) to enhance sample purification, as described in a previous study.^32^ From G6 onward, DNA extraction was performed from 15 mL of each replicate independently. For generation 10, DNA and RNA were coextracted from each individual serum bottle using the RNeasy PowerMicrobiome Kit (Qiagen, Germany) according to manufacturer’s instructions. Nucleic acid quality and quantity were assessed using a NanoDrop2000 spectrophotometer (Thermo Fisher Scientific, Waltham, MA, USA) and a Qubit fluorometer (Invitrogen, Carlsbad, CA, USA). Total RNA integrity was further evaluated using an Agilent 2100 Bioanalyzer with RNA 6000 Nano reagents and chips (Agilent Technologies, Santa Clara, CA, USA), yielding RNA Integrity Numbers (RIN) between 6.4 and 8.3. Ribosomal RNA depletion was performed using the FastSelect—5S/16S/23S Kit (Qiagen, Germany). DNA and RNA libraries were prepared at the DiBio Sequencing Facility of the University of Padua (Padova, Italy) using the Illumina DNA Prep and the TruSeq Stranded Total RNA kits, respectively, with IDT for Illumina DNA/RNA UD Indexes (Illumina Inc., San Diego, CA, USA). Paired-end sequencing was performed on the NovaSeq 6000 platform (Illumina Inc., San Diego, CA, USA). Raw sequencing data were deposited in the National Center for Biotechnology Information (NCBI) Sequence Read Archive under BioProject accession number PRJNA1454293, with individual data sets associated with their respective BioSample records (SAMN57297198 to SAMN57297206).

### 2.5 Metagenomic and Metatranscriptomic Data Analysis

Genome-centric metagenomics and metatranscriptomics were performed by adapting previously described protocols, as well as the parameters used for the bioinformatic analysis.^33,34^ Raw reads were quality filtered using fastp v0.24.1.^35^ High-quality metagenomic reads from all samples were coassembled using SPAdes v4.0.0 ^36^ and Metagenome-Assembled Genomes (MAGs) were reconstructed using a multitool binning approach, followed by dereplication with Binette v1.1.1.^37^ Taxonomic assignment and relative abundance (RA) were determined using GTDB-Tk v2.4.1 ^38^ (GTDB r226) and CheckM v1.2.3,^39^ respectively. Gene prediction was performed with Prodigal v2.6.3,^40^ and functional annotation was conducted using eggNOG-mapper v2.1.12.^41^ Metabolic pathways were manually reconstructed by interrogating the Kyoto Encyclopedia of Genes and Genomes (KEGG),^42^ and module completeness was defined according to the KEGG rules implemented in the KEMET software.^43^ To evaluate nonstandard syntrophic pathways not yet officially categorized in the KEGG database, custom modules (M10001, M10002, M10003) were defined (Supplementary Table S1). Specifically, the WL/GCS (M10001) and butyrate oxidation pathway (M10002) were implemented based on a previous study.^44^ Carbohydrate-active enzymes (CAZymes) were investigated using dbCAN (v12.0)^45^ databases to estimate the polymer-degrading potential of the species. Phylogenetic relationships were inferred using PhyloPhlAn v3.1.68,^46^ and the resulting tree was visualized with iTOL v7.4.1.^47^ RNA-seq reads were aligned to the panel of reconstructed MAGs using Strobealign v0.16.0.^48^ HTSeq v2.0.9 ^49^ was utilized to count the number of reads mapping per gene in stranded mode, using the GFF files generated by Prodigal^40^ as input. Transcripts per million (TPM) were obtained through normalization of counts by gene length and sequencing depth. RA and TPM were computed for each replicate. Data shown in this study represent the mean values calculated across these data sets. Functional redundancy at the community level was quantified as previously described,^50^ based on functional profiles inferred using MICROPHERRET AD-specific models.^51^ Redundancy at the level of the individual predicted functions was also estimated as the fraction of the community performing each function, i.e., proportion and as RA, calculated as the sum of the abundances of those microorganisms. Functional redundancy analysis was performed by considering only the MAGs with a completeness of ≥70%.

## 3 Results and Discussion

### 3.1 Impact of Progressive Ammonia Loading on Methane Production and Volatile Fatty Acid Fluctuations

A long-term enrichment experiment was conducted to evaluate the impact of increasing FAN concentrations on the structure and activity of an OFMSW-degrading microbiome. The microbial community was subjected to a stepwise increase in this selective pressure over ten generations (G1–G10), spanning a 240 day period (Supplementary Table S2). FAN levels were raised more than four times, from 350 mgN L^–1^ at G1 to 1500 mgN L^–1^ at G10 and were maintained within the target range by adjusting pH to 8.0 ± 0.1 every 3 days.

During early stages (G1–G4), pH exhibited a consistent pattern, with initial drops to 6.5 followed by later stabilization around 8 (Figure 1A). From G5 onward, acidification persisted throughout each generation, indicating progressive system destabilization.

**Figure 1:**
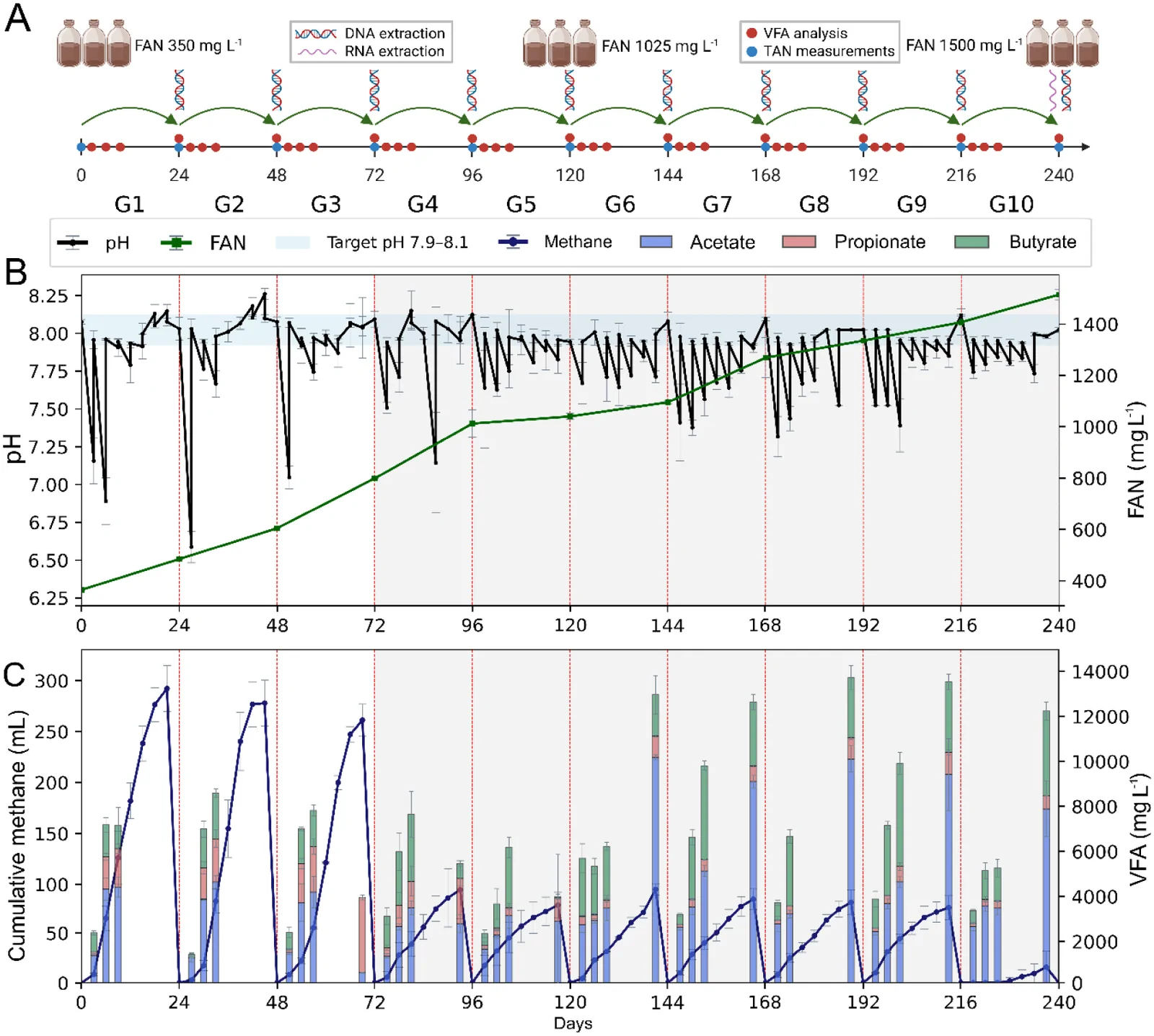
Biochemical monitoring of OFMSW-degrading microbiome over increasing NH_3_. (A) Schematic representation of the acclimation experiment. (B) CH_4_, pH, TAN, and VFA were monitored across generations. Red vertical lines mark generation boundaries, while the gray shaded area (from G4 onward) indicates the onset of inhibition based on CH_4_ production. FAN trend (green), reaching 1560 mgN L^–1^ at the end of the experiment, and pH (black) dynamics to maintain values between 7.9 and 8.1 are shown. (C) Cumulative CH_4_ production per generation (navy blue), with histograms showing VFA production subdivided into acetate, propionate, and butyrate.

Cumulative CH_4_ production remained stable up to G3, yielding approximately 275 mL over 24 days. A marked shift emerged at G4 (780 mgN L^–1^ FAN), where a reduction in CH_4_ yield exceeding 50% was observed (Figure 1B). Despite the increasing inhibitory environment, CH_4_ production remained constant up to 1393 mgN L^–1^ FAN (G9), stabilizing around 100 mL, demonstrating considerable microbial resistance. These results confirm the capacity of AD microbiomes to adapt to FAN levels well above the typical process limits of 500–700 mgN L^52^ ^–123,^. Finally, in G10, the system reached a terminal state, where a sharp decline in CH_4_ production was observed, together with a prolonged lag phase prior to recovery, indicative of severe NH_3_-induced inhibition. This behavior is coherent with the inhibitory thresholds reported in previous studies,^21^ where methanogenic activity was completely suppressed at FAN levels above 1450 mgN L^–1^.

Further insight into the bioconversion dynamics was obtained from CO_2_ and H_2_ rates of consumption and synthesis. Cumulative CO_2_ production mimicked the CH_4_ trajectories, reaching about 65 mL during G1 to G3 and subsequently declining to an average of 35 mL by G9. In contrast, H_2_ remained fully consumed with no detectable accumulation until G6 (Figure S1, Supporting Information). From G7 onward, the H_2_ concentration increased markedly, suggesting that fermentative microorganisms present in the selected microbiota can tolerate elevated FAN levels better than methanogens. Previous studies have already hypothesized that this could be linked to the thermodynamic constraints and low energy yield of methanogenesis, combined with the higher metabolic redundancy and adaptability of bacterial communities.^2,53^ As H_2_ production exceeded its consumption, the resulting increase in H_2_ partial pressure likely inhibited syntrophic acetate-oxidizing bacteria (SAOB), as elevated H_2_ levels make acetate oxidation thermodynamically unfavorable.^54^

These dynamics were closely reflected in the VFA profiles. In early generations, acetate, propionate, and butyrate accumulated transiently. Concentrations measured at the last time point of each generation were lower than initial values, indicating efficient syntrophic conversion (Figure 1B). However, from G6 onward, acetate accumulated at the end of each generation, reflecting the increasing inability of the methanogenic fraction of the community to process intermediate metabolites under high NH_3_ loads.^55^ This behavior is consistent with a shift from acetoclastic methanogenesis to a SAO-hydrogenotrophic pathway, driven by the higher sensitivity of acetoclastic methanogens and the greater tolerance and metabolic flexibility of hydrogenotrophic counterparts.^15,26^ Although this syntrophic configuration prevents complete process failure, its unfavorable energetics explain why acetate persisted in the later generations (Figure 1B). Propionate concentrations declined from G5 onward and did not exhibit accumulation during the period preceding H_2_ buildup (from G7 onward), contrasting with patterns typically reported under NH_3_-induced process imbalance.

### 3.2 Taxonomic Succession Drives the Replacement of Acetoclastic Methanogens by Ammonia-Tolerant Hydrogenotrophs

Microbiome dynamics were monitored to evaluate the impact of increasing FAN concentrations on community structure and functionality. The coassembly resulting from samples of all ten generations covered a total of 240 Mb, with an average alignment rate of reads to the assembly equal to 98% (Supplementary Table S3). The high alignment fraction indicates that the community was nearly entirely represented, which allowed accurate metabolic reconstruction during downstream analysis. Metagenomic reconstruction of the microbiome recovered 71 MAGs, of which 51 were high-quality (completeness ≥90%; contamination ≤5%) and 20 were medium-quality (50% ≤ completeness ≤ 90% and 5% ≤ contamination ≤ 10%) (Figure 2A), which on average had an alignment rate of ≃91% across all generations (Supplementary Table S4). MAGs included 62 bacterial and 9 archaeal lineages distributed across 11 taxonomic classes (Supplementary Table S5). The community was dominated by members of the class Clostridia (≃59%), consistent with their specialized role in the hydrolysis and fermentation of the complex organic substrates present in OFMSW.^56^

**Figure 2:**
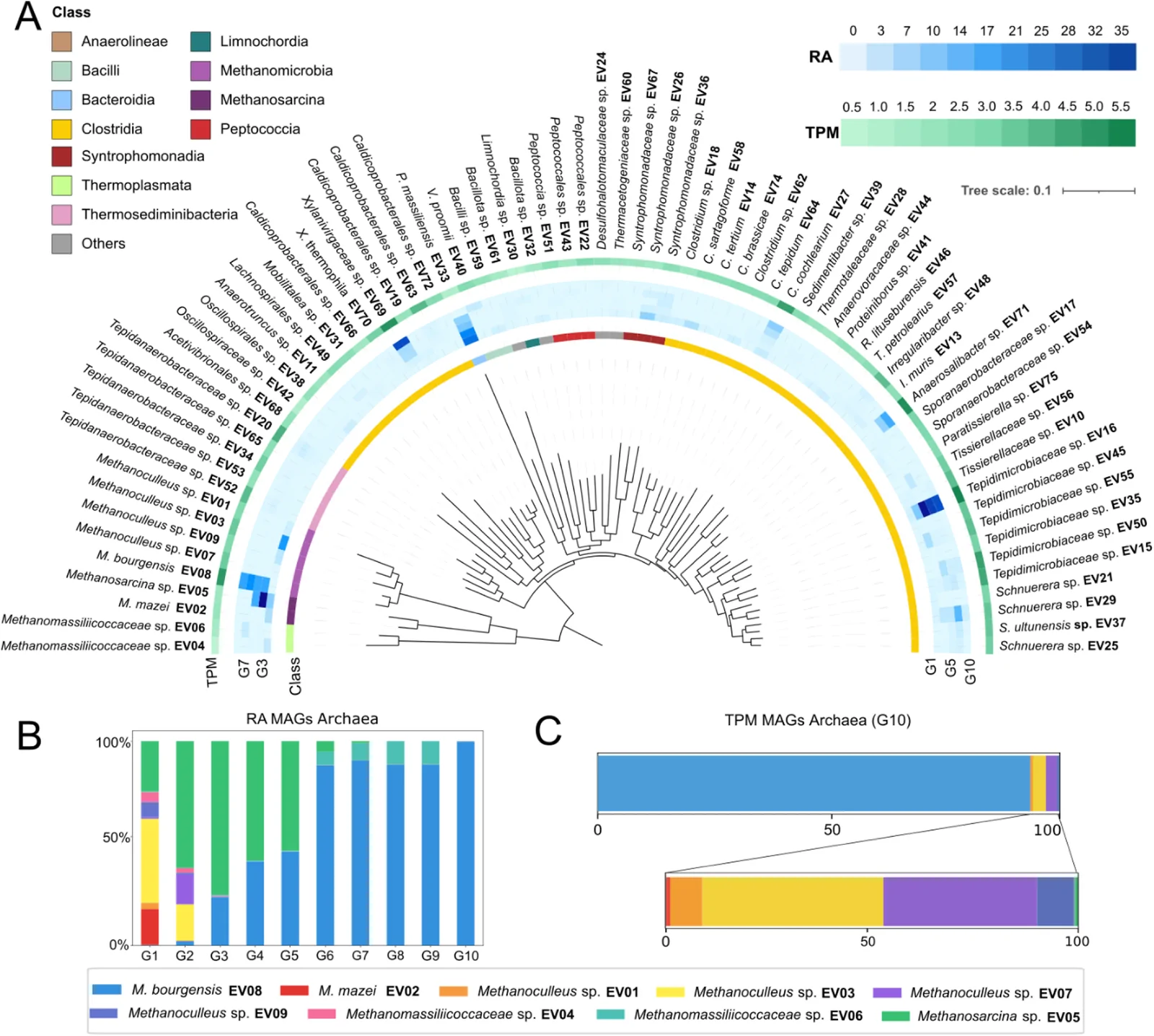
Microbiome reconstruction of an OFMSW-degrading community. (A) Overview of the microbial community composition across the selected generations. MAGs are arranged according to their phylogenetic relationships, with class, relative abundance (RA), and TPM shown as outer rings. (B) Changes in the RA of archaea across generations. (C) Transcriptional activity reported as TPM values of each archaeal species in generation 10, excluding *M. bourgensis* EV08.

At the end of the first generation (G1), the microbiome displayed a heterogeneous composition, reflecting the initial complexity of the inoculum. A progressive simplification was observed over generations, which was likely driven mainly by NH_3_-induced stress. While the transition from a heterogeneous industrial environment to a standardized laboratory growth medium undoubtedly induced profound community restructuring, this shift stabilized rapidly. Given the strong selective pressure posed by the uniform substrate composition, the initial activation phase, and the fact that the community had already undergone a 1:100 dilution by G1, any generalist species incapable of growing on BA medium would have faced rapid washout, dropping to negligible abundances during the earliest stages. Consequently, adaptation to the modified growth medium played a minor role in the initial phases. This continuous selection is clearly illustrated by the gradual, multigenerational abundance trajectories of the dominant species, rather than abrupt, immediate extinctions.

The archaeal fraction of the community initially exhibited high functional redundancy, with nine taxa representing all the methanogenic metabolic pathways (hydrogenotrophic, acetoclastic, and methylotrophic). Based on the predicted metabolism of the archaeal MAGs, in G1, hydrogenotrophic methanogenesis was dominant, representing approximately 21% of the entire community, followed by the acetoclastic route (≃11%) and, to a lesser extent, the methylotrophic one (2.6%). From G2, *Methanosarcina* sp. EV05 became the dominant methanogen, reaching 36% of the total community by G3, while the representatives of the hydrogenotrophic route found in G1 decreased to less than 1%. Given the metabolic profile of the *Methanosarcina* genus, it presumably contributed to methanogenesis primarily via the acetoclastic route, although its metabolic versatility does not allow alternative methanogenic pathways to be excluded. However, as FAN concentrations increased, a clear successional shift occurred. Starting from G3, *Methanoculleus bourgensis* EV08 RA progressively increased, concomitantly with a gradual decline of *Methanosarcina* sp. EV05. This successional pattern is consistent with previous observations in NH_3_-stressed digesters, where increasing FAN concentrations progressively suppress acetoclastic methanogenesis and favor hydrogenotrophic methanogens.^23^ Although *Methanosarcina* spp. are generally more NH_3_ tolerant than *Methanosaeta* spp., they can still be inhibited under severe stress,^57^ whereas *Methanoculleus* spp. are frequently reported as dominant methanogens in NH_3_-adapted communities. From G6 onward, at FAN equal to or higher than 1026 mgN L^–1^, *M. bourgensis* EV08 became the dominant methanogen (RA > 15%), while *Methanosarcina* sp. EV05 RA dropped to 1%, disappearing completely by the end of G9 at a FAN of 1393 mgN L^–1^. This point marked a transition toward predominantly hydrogenotrophic methanogenesis (Figure 2B) and coincided with the onset of acetate accumulation and a sustained decline in pH (Figure 1A), highlighting the metabolic bottleneck created by the suppression of acetate-consuming archaea.

Parallel to the archaeal shift, the bacterial community also underwent a progressive and profound restructuring. The most abundant bacterial taxa in G1 included *Proteiniphilum massiliensis* EV33, *Syntrophomonadaceae* sp. EV26, and *Clostridium sartagoforme* EV58 (Figure S2, Supporting Information), with RA values of 22.4%, 6.5%, and 4.1%, respectively. *P. massiliensis* EV33 and *C. sartagoforme* EV58 are putatively involved in the degradation of proteinaceous and cellulosic fractions of the OFMSW, thus providing the metabolic foundation for downstream methanogenesis.^58^ In particular, *P. massiliensis* EV33 appears to preferentially target protein-rich substrates;^59^ however, previous studies have highlighted its capacity to degrade complex polysaccharides, including cellulose and xylan.^60^ This broad substrate range supports its role as a key primary degrader within the community. In parallel, *Syntrophomonadaceae* sp. EV26 was likely involved in butyrate oxidation, potentially acting in syntrophic association with methanogens.^61^ These early stage generalists were gradually replaced by a consortium of NH_3_-tolerant specialists (Figure 2A, Supporting Information Table S6). Similar taxonomic restructuring has been reported in NH_3_-adapted digesters, where elevated FAN concentrations act as a strong selective pressure favoring populations capable of maintaining metabolic activity under osmotic and energetic stress.^62^ By G10, the community was dominated by *Tepidimicrobiaceae* sp. EV16 and *Xylanivirgaceae* sp. EV69, reaching an RA of >25%, followed by *Clostridium cochlearium* EV27 and *Anaerosalibacter* sp. EV71, which averaged an RA above 6.5%. In particular, the family *Xylanivirgaceae* comprises cellulose and xylan degraders isolated from ammonium-rich environments.^63^ Although *Xylanivirgaceae* sp. EV69 remained at a low RA (<9%) during most of the experiment, its sharp increase in G10 suggests its ability to occupy a cellulosic degradative niche generated under extreme FAN levels (Supporting Information Table S6). Conversely, the enrichment of halotolerant degraders, such as *Anaerosalibacter* sp. EV71, likely reflects the adaptation to increased ionic strength associated with progressive NH_4_Cl supplementation. Members of this genus have been recovered from saline and anaerobic environments and possess physiological traits associated with tolerance to osmotic stress, which may have provided a competitive advantage under the combined NH_3_ and salt pressures imposed during the enrichment process,^64,65^ suggesting that the community restructuring at later stages was driven by a combination of NH_3_ and osmotic stress.

Gene expression profiles at G10 confirmed a strong correspondence between RA and transcriptional activity, with dominant taxa also representing the most metabolically active members of the community (Supporting Information Table S7). *Tepidimicrobiaceae* sp. EV16 exhibited the highest average transcriptional activity (372 889 TPM), followed by *Anaerosalibacter* sp. EV71, *Xylanivirgaceae* sp. EV69, and *C. cochlearium* EV27 (Figure 2A). Notably, *Tepidimicrobiaceae* sp. EV50 showed sustained transcriptional activity comparable to that of *C. cochlearium* EV27, despite its low abundance (RA 1.7%). *M. bourgensis* EV08 expression levels averaged 91,797 TPM (Figure 2A), representing 93.5% of the total archaeal activity and thus showcasing how this archaeon outclasses the rest under challenging NH_3_ concentrations (Figure 2C). This resilience is consistent with previous reports identifying *Methanoculleus* *spp.* as key archaeal populations in NH_3_-adapted digesters.^18^ Beyond the intrinsic advantage of hydrogenotrophic methanogenesis under inhibitory conditions, their persistence has been associated with enhanced tolerance to NH_3_-induced stress and close syntrophic interactions with acetate-oxidizing bacteria.

### 3.3 Identification of Genomic Blueprints of Resilient Syntrophs

Functional annotation of the recovered MAGs was performed to reconstruct the putative metabolic network. From this network, putative syntrophic interactions potentially sustaining the community survival under extreme NH_3_ stress were inferred. KEGG module analysis of dominant taxa (RA > 1%) revealed that core energy metabolism and carbon fixation pathways were consistently present across different functional microbial groups (Figure 3A, Supplementary Table S8). The Wood–Ljungdahl (WL) route (M00377), classically associated with reductive acetogenesis, appeared to operate bidirectionally in this system. During early generations, this pathway likely supported acetate production. Under increasing selective pressure, however, the pathway was potentially redirected toward SAO, compensating for the progressive inhibition of the acetoclastic methanogenesis route. This shift was particularly evident in generations G8 and G9, where Tepidanaerobacteraceae lineages, known to use the pathway in the oxidative direction,^66^ were enriched. Consistent with the H_2_ dynamics reported above, the rise of H_2_ partial pressure from G8 onward likely caused a partial SAO inhibition, limiting acetate conversion and contributing to its accumulation in the later stages.^67^

**Figure 3:**
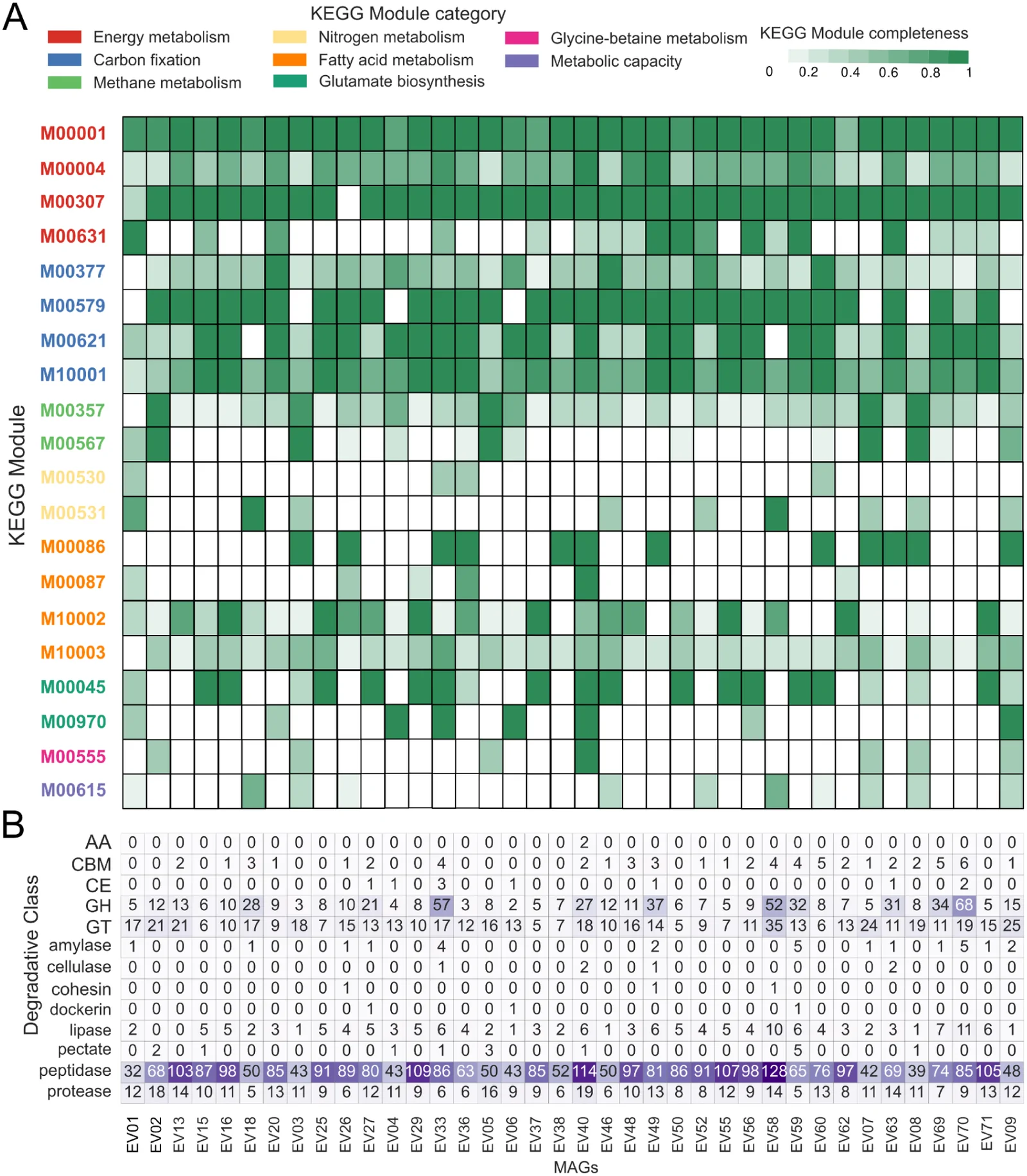
Functional annotation analysis of the dominant microbiome fraction. Genomic potential for central metabolism and substrate degradation across the MAGs recovered. (A) Completeness of selected KEGG modules across major functional categories, including methanogenesis, carbon fixation, VFA degradation, and osmoprotectant biosynthesis. (B) Gene copy numbers per MAG for functions related to protein and cellulose degradation. Only putative metabolic roles of MAGs having relative abundance (RA) >1% were explored. Abbreviations are provided for some of the classes of CAZymes covered, including AA, auxiliary activities; CBM, carbohydrate-binding modules; CE, carbohydrate esterases; GH, glycoside hydrolases; GT, glycosyl transferases.

Notably, several dominant taxa encoded a noncanonical variant of the WL pathway. For instance, members of the *Tepidimicrobiaceae* lineage lacked the canonical carbonyl branch (Figure 3A, Supporting Information Table S9). This structural variation indicates that adaptation to high FAN concentrations involved not only taxa replacement but also specific genomic characteristics enabling alternative carbon-conversion routes in harsh conditions. In these organisms, the glycine cleavage system (GCS; M00621) is predicted to act as a metabolic bypass that channels acetate through glycine. The subsequent cleavage of glycine yields CO_2_ and a reduced C1 unit, which can be further processed to generate H_2_ (Figure 3A).^68^ This combined WL/GCS pathway is not reported in the KEGG database and was represented as a custom module (M1001) for acetate oxidation,^44^ which is widely distributed among bacterial MAGs. Notably, *Tepidimicrobiaceae* sp. EV16, *Schnuerera* sp. EV29, and *Anaerosalibacter* sp. EV71 lacked the typical enzymatic strategies for acetate activation, including both phosphate acetyltransferase (*pta*) and acetyl-CoA synthetase (*acs*) genes but were dominant from G5 onward, reaching 45%, 12%, and 13% RA, respectively (Supporting Information Table S9). Rather than reflecting misannotation, this pattern suggests the presence of alternative enzymatic strategies for the utilization of acetate. Consistent with this interpretation, members of the genus *Schnuerera* have been reported to compensate for the absence of *pta* through alternative enzymes such as butyryl-phosphate transferase (*ptb*)^69^ (Figure S3, Supporting Information). Importantly, *ptb* was also encoded in all of the phylogenetically related MAGs, including *Tepidimicrobiaceae* lineages and *Anaerosalibacter* sp. EV71, indicating a conserved metabolic feature. Together, these findings support the existence of a specialized and noncanonical mechanism sustaining acetate oxidation in the absence of the known pathways, including both the canonical WL pathway and the alternative WL/GCS route. While the involvement of SAO in NH_3_-adapted digesters is well-established, the coordinated genomic and transcriptional evidence supporting the contribution of the alternative WL/GCS module in several enriched taxa represents a less explored aspect of acetate metabolism under severe NH_3_ stress.

Beyond acetate turnover, VFA metabolism provided further insight into the potential syntrophic interactions within the community. Canonical propionate degradation pathways (M10003)^44^ were incomplete across all MAGs analyzed, indicating the absence of putative syntrophic propionate-oxidizing bacteria. Interestingly, the only MAG exhibiting strong genomic potential for propionate production was *P. massiliensis* EV33 (Figure S4, Supporting Information), whose disappearance after G5 temporally coincided with the marked decline in propionate concentrations (Figure 1B), supporting the hypothesis that reduced production, rather than impaired oxidation, underlies the observed VFA profile. In contrast, butyrate metabolism (M10002)^44^ was more widely represented, with complete degradation pathways identified in *Tepidimicrobiaceae* sp. EV16 and EV55, *Schnuerera* sp. EV25 and EV29, *Clostridium* sp. EV62, *Anaerosalibacter* sp. EV71 (Figure S4, Supporting Information). Although the pathway in *Syntrophomonadaceae* sp. EV26 is missing the first enzyme, comparative genomic analysis with *Syntrophomonas wolfei*,^70^ a well-characterized butyrate oxidizer, revealed a conserved genomic organization. In this configuration, the key pathway genes are clustered within an operon that includes a putative acetyl-CoA hydrolase, which likely fulfills the role of the missing initial step. Notably, this same configuration was identified in *Syntrophomonadaceae* sp. EV26 and *Syntrophomonadaceae* sp. EV36, where the absence of a clearly annotated first enzyme is compensated for by the presence of this functionally inferred protein.

The hydrolytic and fermentative guild also underwent a severe rearrangement. During early stages (G1–G5), organic matter degradation was predominantly mediated by a diverse consortium of microbes, including *Irregularibacter muris* EV13, *Clostridium* sp. EV18, Lachnospirales sp. EV49, *C. sartagoforme* EV58, *Caldicoprobacterales* sp. EV63, and *Xylanivirgaceae* sp. EV69. Among these, *P. massiliensis* EV33 emerged as the key hydrolytic bacterium, encoding both peptidases and glycoside hydrolases (Figure 3B), alongside the functional module for acetate production (M00579). This genetic repertoire suggests that it may operate as a flexible “metabolic bridge” linking hydrolysis and SAO.^71^ However, at later stages (G8–G10), this early community was progressively replaced by an NH_3_-tolerant consortium. Specifically, *Xylanivirgaceae* sp. EV69 emerged as an alternative hydrolytic degrader of xylose-rich cellulosic substrates, as its genome encodes several glycoside hydrolases, a function already characterized for this bacterial family in previous studies.^63^ In addition to metabolic rewiring, osmoprotection emerged as a critical adaptive trait (Supplementary Table S10). Most NH_3_-tolerant members (i.e., *Tepidimicrobiaceae* sp. EV16, *M. bourgensis* EV08, *Anaerosalibacter* sp. EV71) appeared to rely on glutamate accumulation, holding *hutH*, *hutU*, *hutG*, *putA,* and *putB* genes for conversion of histidine or proline into glutamate.^72^ Conversely, *Virgibacillus promii* EV40 was the only species encoding the biosynthetic machinery for glycine-betaine (i.e., *gbsA* and *gbsB*), which, once synthesized, can help balance cellular osmotic pressure and protect proteins from denaturation.^19^ Such diversification suggests niche specialization in osmoprotectant strategies. The dominant *M. bourgensis* EV08 was possibly adopting a third strategy, thanks to its encoded beta-lysine N6-acetyltransferase (*ablB*). In archaea, this protein is induced under salt stress and the product, N6-acetyl-beta-l-lysine, serves as a compatible solute, conferring high salt resistance to the producing organisms.^73,74^ In addition to this, the presence of an ABC-type quaternary amine transporter (ProVWX) suggests that the archaeon can mediate the high-affinity uptake of glycine-betaine from the extracellular space,^75^ thereby contributing to osmotic stress mitigation. These mechanisms combined are likely further supported by the activity of the Trk/Ktr potassium uptake system (*trkA*, *trkH*), which together enhances intracellular osmotic balance. Gene count analysis also revealed that *M. bourgensis* EV08 encodes a higher number of copies of these stress-related genes compared to other methanogens (), potentially enabling increased transcriptional capacity and contributing to its resilience under elevated NH_3_ conditions. Moreover, dominant microorganisms at the later stages expressed a greater variety of NH_3_-resistance genes compared to their counterparts in G1 (Figure S5, Supporting Information). Collectively, although they need metatranscriptomic confirmation, these results indicate that adaptation to high-NH_3_ conditions involves a coordinated metabolic reorganization toward SAO and hydrogenotrophic methanogenesis.

### 3.4 Transcriptional Activation of Ion Transporters and Osmoprotectant Systems Characterizes the Microbial Response at High FAN

To identify the metabolic function remaining active under inhibitory NH_3_ conditions, transcriptomic analysis was performed on the G10 community, focusing on MAGs with an RA above 1% and individual genes with expression levels >10 TPM in at least one species (Figure 4). Expression profiles corroborated the genomic evidence of metabolic rewiring within the WL pathway. Tepidanaerobacteraceae lineages (EV20 and EV52) primarily expressed canonical WL genes. In contrast, several putative SAOB, including *Tepidimicrobiaceae* members (EV15, EV16, EV50, and EV55) and *Schnuerera* sp. EV29, *Schnuerera ultunensis* EV37, and *Anaerosalibacter* sp. EV71, showed strong transcriptional activity of GCS-related genes (i.e., *grdE*, *grdB*, *gcvPA*, *gcvPB*, *gcvT*, *gcvH*) (Figure 4A, Figure S6A, Supporting Information). All of these species also exhibited medium-high expression of *ptb*, supporting the putative role of this gene as a key enzymatic driver of acetate oxidation in the absence of canonical *ackA* + *pta* or *acs* genes. Importantly, *Tepidimicrobiaceae* sp. EV16 and EV55, *Schnuerera* sp. EV29, *S. ultunensis* EV37, and *Anaerosalibacter* sp. EV71, in addition to sustained expression of WL/GCS pathway genes, exhibited a complete and actively transcribed butyrate degradation pathway (Figure 4B, Figure S6B, Supporting Information). This coexpression pattern suggests a high degree of metabolic versatility, potentially enabling these organisms to contribute to both butyrate oxidation and downstream acetate turnover. Rather than indicating a fully self-contained pathway, these functions are likely integrated within broader syntrophic interactions, supporting carbon flux from butyrate toward H_2_ and CO_2_ under NH_3_ stress conditions.

**Figure 4:**
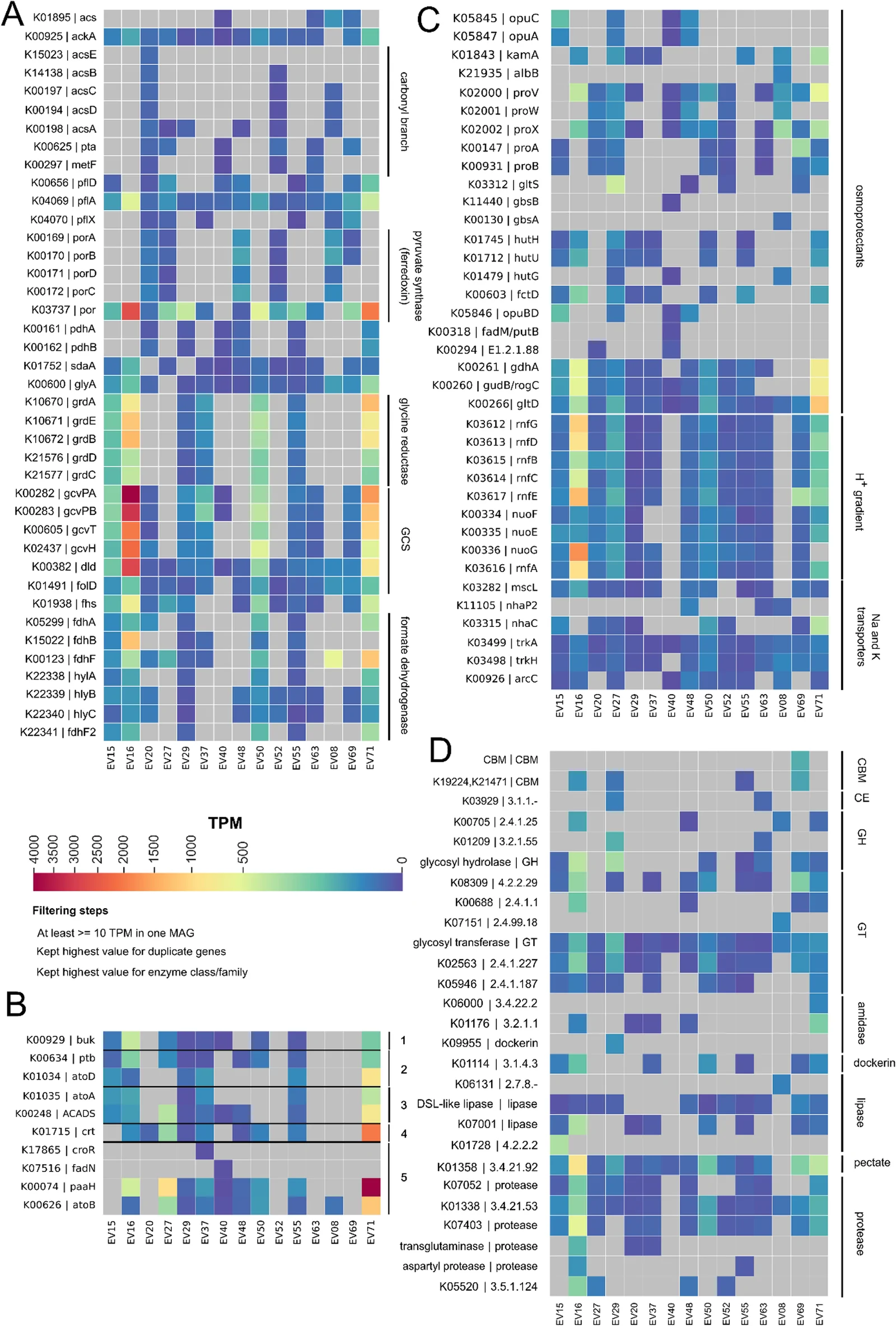
Gene expression profiles across key metabolic functions at G10. Expression (TPM) of key functional genes across the transcriptionally active MAGs. All panels share the same TPM color scale, gray cells indicate genes below the threshold (<10 TPM in every MAG) or absent. (A) Genes encoding enzymes of the Wood–Ljungdahl (WL) pathway and its alternative branch mediated by the glycine cleavage system (GCS). (B) Genes of the butyrate oxidation pathway, resolved into individual enzymatic steps (1–5). (C) Genes encoding proteins involved in mechanisms of NH_3_ resistance, including ion transport systems (K^+^ and Na^+^), proton gradients, and genes involved in the biosynthesis and uptake of osmoprotectants. (D) Distribution of degradative enzyme genes across MAGs, focusing on protein- and cellulose-associated functions as defined by CAZy.

The molecular basis of NH_3_ tolerance relied on the expression of specialized stress response systems. As un-ionized NH_3_ diffuses across the cell membrane and becomes protonated to NH_4_^+^ inside the cell, cytoplasmic pH and osmotic balance are disrupted. To counteract these effects, several energy-intensive homeostatic mechanisms must be activated. As expected, the transcriptional landscape revealed strong activation of stress response and ion transport systems among dominant taxa, consistent with increased energetic demands for cellular homeostasis under high FAN levels (Figure 4C, Figure S6C, Supplementary Materials, Supplementary Table S11). Specifically, high expression of ATP-dependent potassium transport systems (*Trk/Ktr*) and Na^+^/H^+^ antiporters (*N*ha) was coupled with the upregulation of key respiratory complexes (i.e., *rnfA*, *rfnB*, *rfnC,* and *nouG*). These complexes’ function, respectively, as a membrane-bound ferredoxin oxidoreductase that translocates ions to generate an electrochemical gradient and as an NADH oxidoreductase that contributes to the proton motive force. The robust activation of these bioenergetic systems suggests that critical energetic resources, such as ATP and reducing equivalents, were preferentially allocated to maintaining intracellular pH and osmotic balance over growth.^76,77^ Consistent with this, *Tepidanaerobacteraceae* sp. EV20 and EV52, *Anaerosalibacter* sp. EV71, and *Tepidimicrobiaceae* sp. EV50 and EV15 exhibited strong expression of the Na^+^/H^+^ antiporter (*nhaC*), supporting proton homeostasis, while the Trk/Ktr potassium transport system (i.e., *trkA* and *trkH*) was broadly expressed across the surviving community, including *M. bourgensis* EV08. Together, these systems contribute to intracellular pH stabilization and the regulation of cellular turgor pressure through K^+^ uptake.

Energy conservation under stress conditions appeared to rely on respiratory complexes and electron-bifurcating systems (Figure 4C, Figure S6C, Supporting Information). The genes encoding the NADH–ubiquinone oxidoreductase (Nuo complex) were broadly transcribed, likely contributing to the maintenance of the electrochemical gradient. Additionally, Rnf-type flavoprotein complexes (i.e., *rnfA*, *rfnB*, *rfnC, rfnD, rfnE,* and *rgnG*) were highly expressed in several members of the community, likely coupling ferredoxin reduction with reverse electron transport to sustain the proton motive force required for metabolism under inhibitory conditions.

Compatible solute metabolism emerged as a key adaptive strategy under extreme NH_3_ stress. *V. promii* EV40 encoded the glycine-betaine biosynthetic pathway from choline (Figure 4C); however, this incomplete expression pattern indicates a potentially limited or condition-dependent capacity for glycine-betaine production.^78^ In parallel, the ProVWX transport system, responsible for glycine-betaine uptake, was highly expressed in dominant taxa, such as *Tepidimicrobiaceae* sp. EV16, *Anaerosalibacter* sp. EV71, and the methanogen *M. bourgensis* EV08 (Figure 4C). This suggests that extracellular glycine betaine, potentially derived by partial synthesis, alternative producers, or environmental sources, is actively assimilated by key community members, contributing to osmotic balance and enhancing resilience under high NH_3_ conditions. The uptake of compatible solutes represents an energetically favorable strategy compared with de novo synthesis, particularly under energy-limited conditions. This pattern points to a shared osmoadaptation mechanism operating at the community level. Additional niche-specific strategies were also detected. Glutamate production by *Schnuerera* and *Tepidimicrobiaceae* lineages, actively expressing the *hutH*, *hutU*, and *hutG* genes,^79^ may be coupled with *C. cochlearum* EV27 expression of the Na^+^-dependent glutamate transporter (*gltS*) for extracellular glutamate uptake. Conversely, *M. bourgensis* EV08 exhibited expression of the β-lysine N6-acetyltransferase gene (*albB*), suggesting a primary role in N6-acetyl-β-l-lysine production as an osmoprotectant. In addition, this archaeon expressed the *arcC* gene, encoding a carbamate kinase, which catalyzes the reversible conversion of NH_3_ and hydrogen carbonate into carbamoyl phosphate, potentially contributing to intracellular nitrogen detoxification. These mechanisms indicate that maintaining intracellular turgor pressure is as critical as pH regulation for survival under high FAN conditions. Collectively, this evidence reveals a coordinated transcriptional response in which the energetic cost of NH_3_ detoxification and osmoprotection is mitigated by the activation of specialized energy-conserving pathways operating at the community level.

Despite the substantial energetic burden imposed by NH_3_ stress, the expression of a hydrolytic enzyme repertoire, dominated by proteases, glycosyl hydrolases, and amylases, marks the persistence of degradative activity in the community. *Tepidimicrobiaceae* sp. EV16, together with *Tepidimicrobiaceae* sp. EV50, emerged as the primary degrader, displaying a broad capacity to process both proteinaceous and cellulose-rich components of OFMSW. In addition, alongside these generalists, several taxa expressed more specialized activities: *Tepidimicrobiaceae* sp. EV15 expressed pectate lyase (K01728), highlighting its putative role in the breakdown of structurally complex substrates, while *Xylanivirgaceae* sp. EV69 emerged as the main cellulose degrader through the expression of glycosyl hydrolases. Finally, *Anaerosalibacter* *sp*. EV71 exhibited strong expression of amylolytic enzymes (K01176), alongside peptidases, putatively supporting the degradation of polysaccharides and cellulose-derived compounds. Taken together, these transcriptional patterns should be interpreted within the context of G10, which represents the highest FAN concentration reached during the enrichment process and substantially exceeds levels typically encountered in full-scale digesters. Although this stage was characterized by reduced productivity, CH_4_ production was ultimately restored after an extended lag phase, indicating that the community remained functionally active under severe NH_3_ stress. The concurrent expression of pathways involved in substrate degradation, syntrophic metabolism, methanogenesis, energy conservation, and osmoadaptation suggests that key AD functions were maintained despite operation near the upper inhibitory threshold. Therefore, the observed expression profiles are best interpreted as signatures of resilience and adaptation in a highly stressed but still functioning microbiome, rather than as transcriptional indicators of optimal process performance.

### 3.5 Functional Redundancy across Diverse Taxa Maintains the Core Metabolic Groups Required for AD under Inhibitory Conditions

Analysis of the genomic potential of the community alongside its transcriptional profiles revealed that core community functionality was sustained by four main metabolic guilds: organic matter degraders, SAO, homoacetogens, and methanogens, the latter split into acetoclastic and hydrogenotrophic. During the initial stages of the experiment, CH_4_ production was likely supported by both acetoclastic and hydrogenotrophic pathways, primarily mediated by *Methanosarcina* sp. EV05 and *Methanoculleus* sp. EV03. Within the bacterial community, *P. massiliensis* EV33 emerged as a primary degrader of the OFMSW, with the metabolic capacity to process both the protein and carbohydrate fractions. Consistent with previous studies, it encodes a broad repertoire of cellulose-degrading enzymes, supporting a central role in the breakdown of complex plant-derived polymers, such as xylan (Figure 3B). In addition to its hydrolytic capabilities, this organism harbors the pathway for acetate conversion, indicating a high degree of metabolic versatility. It likely contributed to acetate and propionate production from low-molecular-weight substrates in early stages while retaining the potential to oxidize acetate under syntrophic conditions. This dual functionality positions *P. massiliensis* EV33 as a key metabolic bridge, linking hydrolysis, fermentation, and methanogenesis during the stable phase of the process. On the other hand, *Syntrophomonadaceae* sp. EV26 was likely working downstream as a syntrophic butyrate oxidizer, producing intermediates for the downstream methanogenesis.

As FAN concentrations increased, these early stage colonizers were progressively replaced by more tolerant but functionally equivalent taxa. *Xylanvirgaceae* sp. EV69 replaced *P. massiliensis* EV33 as the main degrader of complex carbohydrates. Beyond EV69, the degradative role was shared among several adapted taxa: the *Tepidimicrobiaceae* lineage (EV16 and EV50) processed both proteinaceous and cellulosic fractions, while *Anaerosalibacter* sp. EV71 and *Tepidimicrobiaceae* sp. EV15 contributed complementary amylolytic and pectinolytic activities. From generation G6 onward, *M. bourgensis* EV08 became the dominant archaeon (Figure 5), in line with the metabolic transition described above. The multiple protection mechanisms encoded in this archaeon ensure an efficient homeostatic response to high NH_3_ concentrations, including osmoprotectant synthesis and the maintenance of intracellular proton and K^+^ homeostasis. Furthermore, this shift was supported by the emergence of a specialized bacterial consortium dominated by members of the *Tepidimicrobiaceae* lineage. In particular, these organisms, alongside *Anaerosalibacter* sp. EV71, have the potential to couple butyrate degradation with the WL/GCS bypass to establish a metabolic bridge between VFA production and acetate consumption when the acetoclastic route is suppressed (Figure 5). Their survival and growth even at later stages were granted by a wide array of strategies to regulate homeostasis, energy conservation, and ATP generation via substrate-level phosphorylation, allowing restoration of a healthy cellular environment during NH_3_ stress. Finally, homoacetogenic bacteria represented a smaller but persistent functional group throughout the experiment. These were primarily affiliated with *Tepidanaerobacteraceae* sp. EV20 and *Thermoacetogeniaceae* sp. EV60, which survived throughout all generations or perished around G6, respectively. In contrast to SAOB, these taxa expressed the canonical WL pathway, fixing CO_2_ and H_2_ for acetate production until the metabolic burden was too high to sustain and the hydrogenotrophic methanogens became the main sink of the carbon fluxes (Figure 5). At the community level, these successions were underpinned by a dynamic reorganization of functional redundancy. Early stages were characterized by high redundancy, ensuring robustness through multiple taxa contributing to overlapping functions. As NH_3_ levels increased, redundancy was progressively streamlined, selecting a few highly specialized and stress-tolerant microorganisms capable of sustaining key bioconversions. Despite the decrease in taxonomic diversity, process stability was maintained as these specialists effectively replaced NH_3_-sensitive populations while preserving core functions. Interestingly, functional redundancy increased again in G10 (Figure S7A, Supporting Information), suggesting a secondary diversification within the adapted community. This behavior could reflect the emergence of alternative strategies to cope with extreme stress conditions such as increasing NH_3_ and salt loads. When focusing on specific functions, cellulose degradation was the most redundant process, involving the highest number of microorganisms and highlighting its central role in sustaining carbon flux in OFMSW degradation (Figure S7B, Supporting Information). Hydrogenotrophic methanogenesis also displayed high redundancy, both in terms of community representation and relative abundance, reinforcing its role as the only viable route under elevated NH_3_ conditions. In contrast, butyrate oxidation and acetoclastic methanogenesis, initially supported by multiple taxa, progressively declined under selective pressure (Supporting Information Table S12). These functions first became restricted to a few specialized organisms and eventually disappeared, reflecting the loss of species that are less competitive or resilient under high NH_3_ stress.

**Figure 5:**
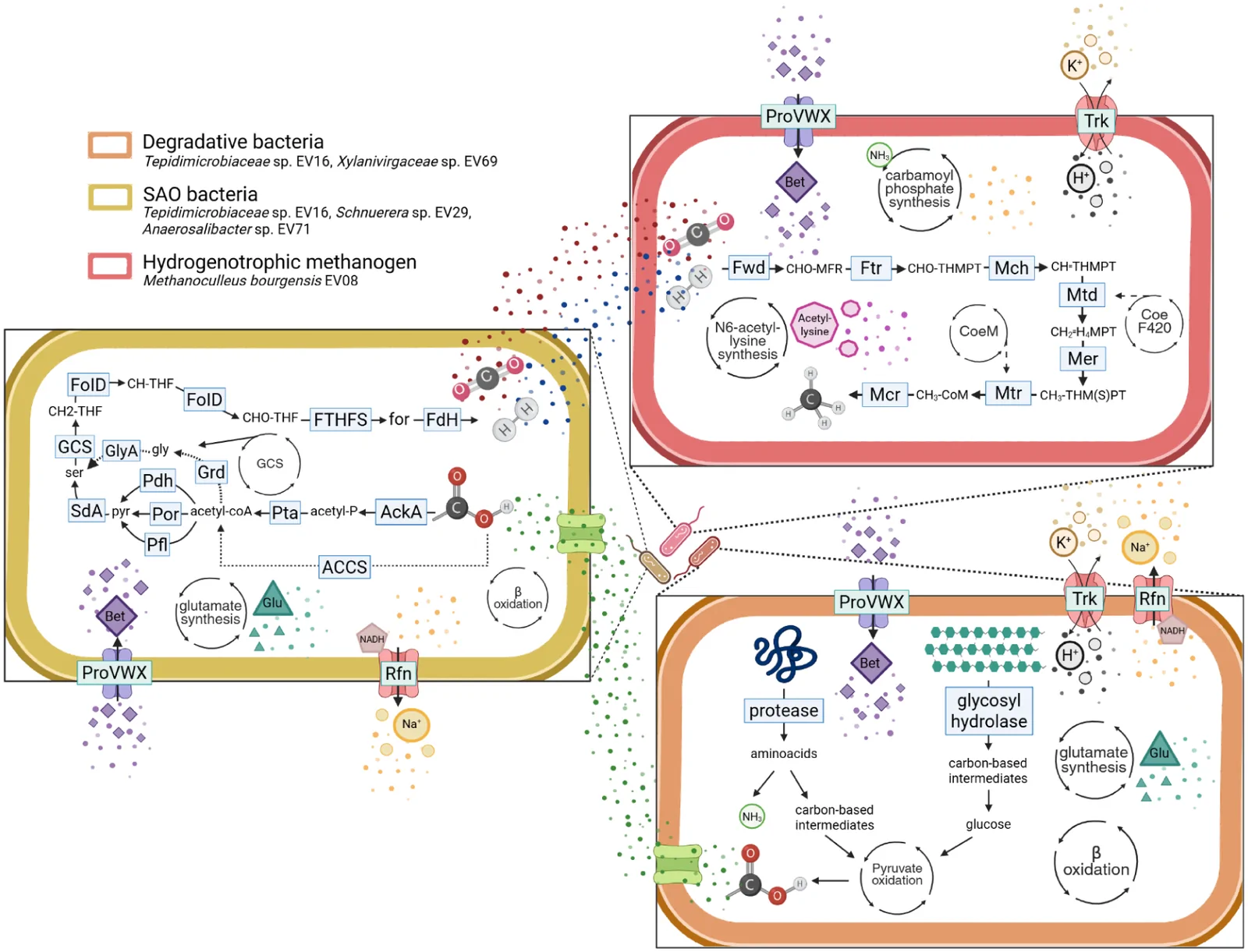
Schematic representation of the main metabolic functions performed by the microbial community at G10. Degradative bacteria (orange) are characterized by a broad repertoire of hydrolytic enzymes, including proteases and glycoside hydrolases, enabling the breakdown of complex organic matter. Syntrophic acetate-oxidizing bacteria (SAOB) (yellow) convert acetate into H_2_ and CO_2_ via the WL/GCS pathway, while hydrogenotrophic methanogens (red) utilize these intermediates for CH_4_ production. Key NH_3_ resistance mechanisms are also highlighted, including glutamate synthesis, glycine betaine uptake, and Na^+^/K^+^ based ion transport systems.

Together, these results suggest that the persistence of AD processes under extreme FAN pressure is governed not by taxonomic stability but mainly by functional redundancy. As NH_3_ concentrations increase, stress-sensitive populations are progressively replaced by metabolically equivalent, yet more tolerant organisms, driving a reorganization of the metabolic network. In this context, the direct acetoclastic pathway is ultimately substituted by SAO coupled with hydrogenotrophic methanogenesis, allowing CH_4_ production to continue despite severe inhibitory conditions. This work delineates the molecular and ecological boundaries of the AD microbiome under intense NH_3_ selection, showing how long-term process resistance emerges from continuous taxonomic succession, supported by a reservoir of functionally redundant microorganisms. The shift toward hydrogenotrophic methanogenesis is underpinned by extensive metabolic rewiring, including the emergence of *Tepidimicrobiaceae* lineages exploiting a noncanonical WL/GCS module and *ptb*-mediated acetate activation. While this genomic plasticity sustains SAO and prevents complete system collapse, the high transcriptional investment required for ion homeostasis and osmoprotection imposes a strict energetic burden, ultimately resulting in reduced CH_4_ yields.

### 3.6 Environmental Implications

Nitrogen-rich feedstocks such as OFMSW, food waste, and livestock manure are increasingly directed to AD, yet NH_3_ accumulation remains one of the main operational constraints limiting the treatment of these streams. Our results show that an OFMSW-degrading community can sustain CH_4_ production at FAN concentrations more than twice the conventional inhibitory range and that this resilience is not associated with the persistence of specific microbial taxa but with the maintenance of core metabolic functions through community reorganization. The specific advance of this work is the demonstration that long-term NH_3_ tolerance in AD is governed by functional redundancy and metabolic rewiring rather than by taxonomic stability: as stress-sensitive populations are lost, metabolically equivalent but more tolerant organisms take over, restructuring the community. These findings translate into several practical levers for operating full-scale digesters fed with nitrogen-rich substrates. The gradual, stepwise increase in FAN applied here was decisive in extending tolerance well beyond the typical inhibitory threshold, indicating that controlled loading and the preservation of a taxonomically diverse inoculum are concrete strategies to build process resilience. Because the shift toward SAO was accompanied by acetate accumulation and a rising H_2_ partial pressure before CH_4_ output declined, monitoring these two parameters provides an accessible early warning signal of impending inhibition, ahead of any drop in biogas. The RA of hydrogenotrophic and acetate-oxidizing taxa may likewise serve as a microbial biomarker of a successfully adapted community. Finally, the observation that the dominant energetic cost under NH_3_ stress lies in osmotic and ion homeostasis rather than substrate degradation points to a directly testable intervention, supplementation of compatible solutes such as glycine betaine, to relieve part of this burden and help sustain CH_4_ yield. More broadly, these results indicate that monitoring functional traits and metabolic guilds may provide more informative process indicators than taxonomic composition alone for the treatment of nitrogen-rich organic waste.

At the same time, these findings should be interpreted in light of their experimental framework. The use of a controlled batch system to select for NH_3_-tolerant microbes with a single inoculum and substrate simplifies the intrinsic variability of full-scale reactors, where continuous operation and fluctuating conditions may alter both the selection dynamics and metabolic responses. In addition, because NH_4_Cl was used to progressively increase TAN concentrations, the ionic strength increased throughout the enrichment process. Therefore, the observed adaptive trajectories likely reflect a combined response to elevated FAN and osmotic stress rather than to NH_3_ stress alone. Moreover, the metatranscriptomic analysis was restricted to the terminal stage to assess the transcriptional activity of key metabolic pathways under the highest FAN concentration reached during the experiment. Finally, pathway reconstruction relies on genomic and transcriptional proxies rather than direct flux measurements. As a result, the actual contribution and directionality of key processes such as SAO and the WL/GCS bypass remain to be quantitatively validated.

These open questions define clear directions for future research. The proposed noncanonical WL/GCS acetate oxidation route and *ptb*-mediated acetate activation should be confirmed through direct approaches such as ^13^C metabolic flux analysis, targeted proteomics, and the isolation of candidate organisms. Continuous-flow reactors are needed to test whether the adaptive trajectory observed in this batch system holds under realistic operating dynamics, while time-resolved meta-omics across the full acclimation period, integrated with genome-scale metabolic modeling and machine-learning-based functional prediction, would resolve the temporal sequence and quantitative contribution of these adaptations.

Within these boundaries, the combined application of long-term community enrichment and genome-resolved meta-omics nonetheless provides a powerful framework for uncovering adaptive trajectories under extreme NH_3_ stress. Although several adaptive responses observed in this study, including osmoprotection, ion homeostasis, and the shift toward hydrogenotrophic methanogenesis, are consistent with mechanisms previously reported in NH_3_-stressed anaerobic digesters, our genome-resolved multiomics approach provides new insights into how these functions are distributed and coordinated within an adapted microbiome. In particular, the integration of metagenomic and metatranscriptomic data revealed a guild-based organization of the community and highlighted a remarkable degree of functional redundancy, whereby key metabolic functions were maintained despite extensive taxonomic turnover. By linking community turnover, metabolic rewiring, and process performance, this study offers a mechanistic basis for identifying critical tipping points and designing targeted strategies to enhance the robustness of AD systems treating nitrogen-rich substrates.

## Supplementary Material





## Notes

Supplementary Figures S1–S7; the cumulative CO_2_ and H_2_ production throughout the experiment, complementing the CH_4_ and VFA dynamics in the main text; details the relative abundance of the metagenome-assembled genomes at each generation, the presence of propionate- and butyrate-degradation genes within the dominant genomes at the terminal stage, and the NH_3_-resistance genes in the early (G1) and adapted (G10) communities; characterizes the dynamics of functional redundancy across generations and metabolic functions, and presents the DNA-normalized gene expression profiles at the terminal stage (PDF)
Supplementary table S1–S12. Table S1: Custom KEGG module to investigate the degradation of the principal VFA: acetate, propionate and butyrate; Table S2: biochemical measurements of generations; Table S3: alignment rates on the assembly; Table S4: alignment rates on MAGs; Table S5: GTDB taxonomic assignment of all MAGs; Table S6: MAGs RA through each single generation; Table S7: RA and TPM of MAGs on G10; Table S8: KEGG module of dominant MAGs (RA > 1); Table S9: WL genes count for dominant MAGs (RA > 1); Table S10: NH_3_-resistance genes count for dominant MAGs (RA > 1); Table S11: methanogenesis gene expression (TPM) in Archaea; Table S12: functional redundancy of MAGs divided by functional classes (XLSX)

## Abbreviations

AD: Anaerobic Digestion
CO_2_: Carbon Dioxide
CH_4_: Methane
FAN: Free Ammonia Nitrogen
GCS: Glycine Cleavage System
H_2_: Hydrogen
MAG: Metagenome-Assembled Genome
NH_3_: Ammonia
OFMSW: Organic Fraction of Municipal Solid Waste
RA: Relative Abundance
SAO: Syntrophic Acetate Oxidation
SAOB: Syntrophic Acetate Oxidizing Bacteria
TAN: Total Ammonia Nitrogen
TKN: Total Kjeldahl Nitrogen
TOC: Total Organic Carbon
TPM: Transcripts per Million
TS: Total Solids
WL: Wood–Ljungdahl
VFA: Volatile Fatty Acid
VS: Volatile Solid
