# Supplementary material for: Adaptive Evolution Reveals Metabolic Plasticity and Functional Redundancy in an Anaerobic Microbiome under Extreme Ammonia Stress
Source: Environ Sci Technol. 2026 Aug 26;60(35):24776–91. doi: 10.1021/acs.est.6c05759 (PMC13564446; doi:10.1021/acs.est.6c05759)
Supplement: Supplementary file 1 [file es6c05759_si_001.pdf]

Adaptive evolution reveals metabolic plasticity and functional redundancy in anaerobic microbiome under extreme ammonia stress

Luca Francescato[a], Gabriele Ghiotto[a,b*], Maria Chiara Valerin[a], Nicola De Bernardini[a], Sofia Fraulini[a], Annalisa Sandon[c], Laura Treu[a], Maria Cristina Lavagnolo[c], Stefano Campanaro[a]

[a] Department of Biology, University of Padova, Via U. Bassi 58/b, 35121, Padova, Italy

[b] Joint Genome Institute, Lawrence Berkeley National laboratory, 1 Cyclotron Road, Berkeley, CA 94720, United States of America

[c] Department of Civil, Environmental and Architectural Engineering, University of Padova, Via Marzolo 9, 35131, Padova, Italy

*Corresponding author:

Gabriele Ghiotto (GabrieleGhiotto@lbl.gov), Joint Genome Institute, Lawrence Berkeley National laboratory, 1 Cyclotron Road, Berkeley, CA 94720, United States of America

Index:

Fig. S1 Cumulative CO_2 and H_2 production throughout the ALE experiment.
Fig. S2 Relative abundance of the MAGs at each generation.
Fig. S3 Propionate and butyrate pathways' genes presence within the dominant MAG at G10.
Fig. S4 Genomic potential of propionate production-related functions across MAGs.
Fig. S5 NH_3-resistance genes presence in G1 and G10 MAGs.
Fig. S6 RNA/DNA normalization of the key metabolic function's expression
Fig. S7 Functional redundancy across generations and for functional groups.

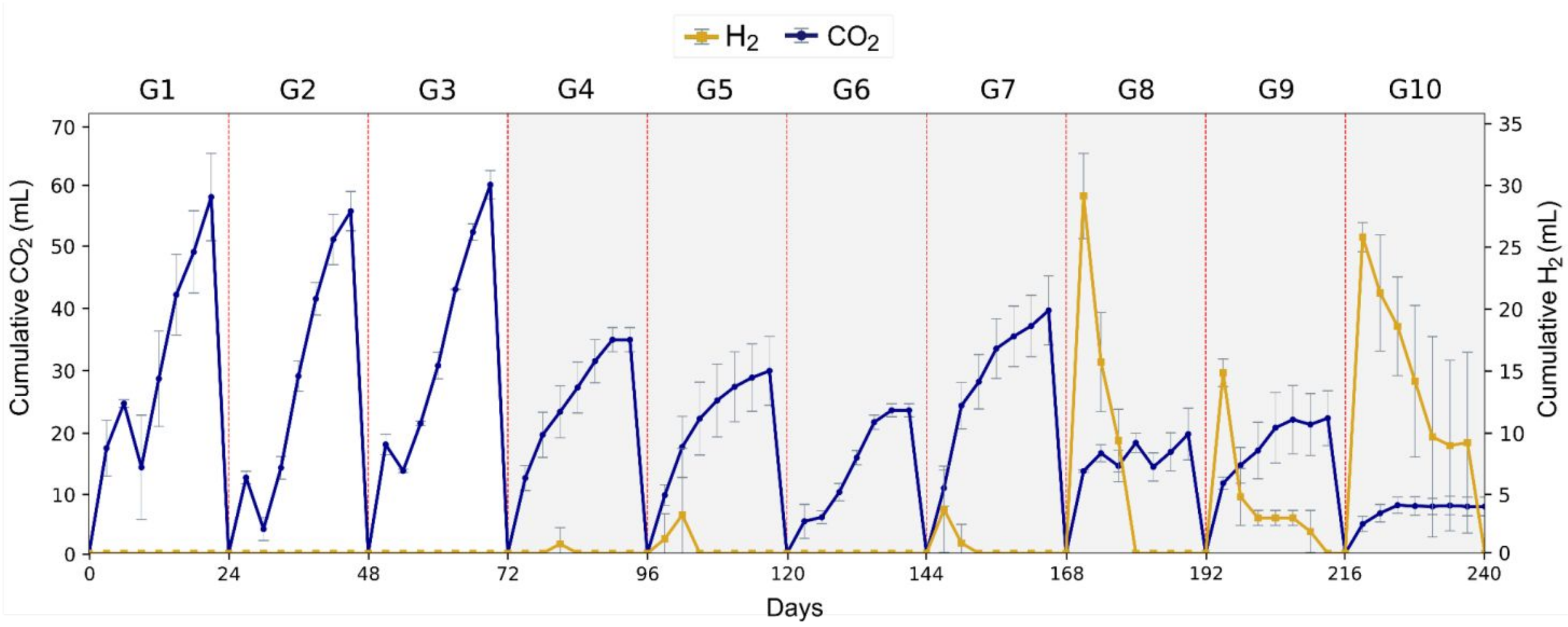

Fig. S1. Cumulative CO_2 (blue) and H_2 (yellow) production at each generation (G1-G10) .

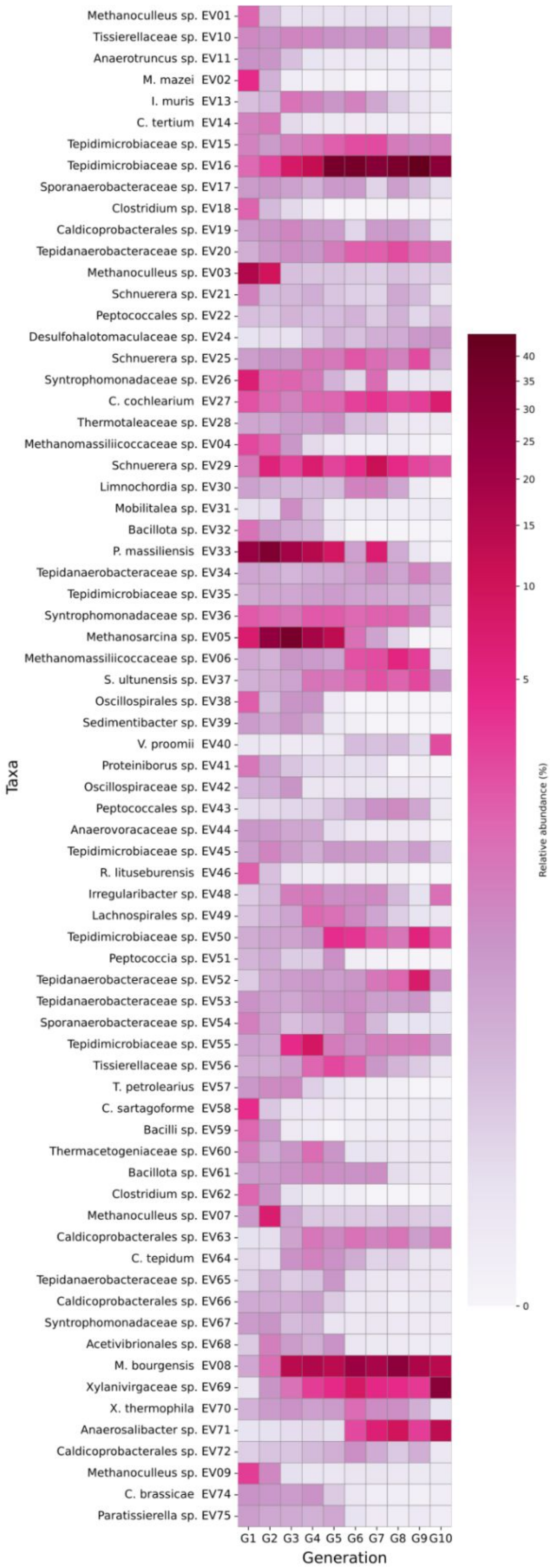

Fig. S2. Heat map presenting the relative abundance (RA) of each reconstructed MAG throughout the generations on a logarithmic scale from 0 to 40. RA for G7-G10 is the average of the three biological replicates.

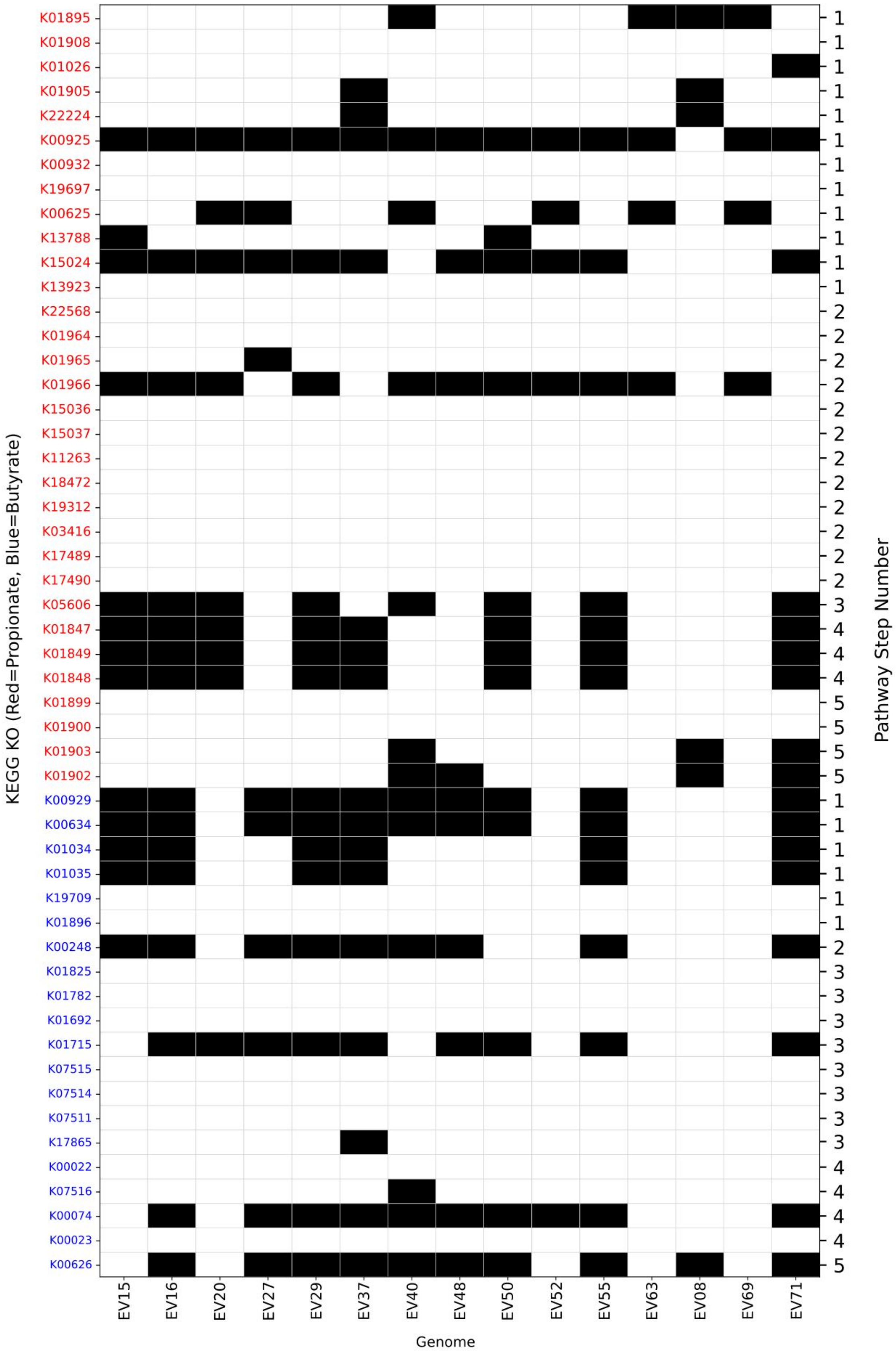

Fig. S3. Presence or absence of KEGG orthologs associated with propionate (red) and butyrate (blue) metabolism across dominant MAGs in G10, organized by pathway step (1-5).

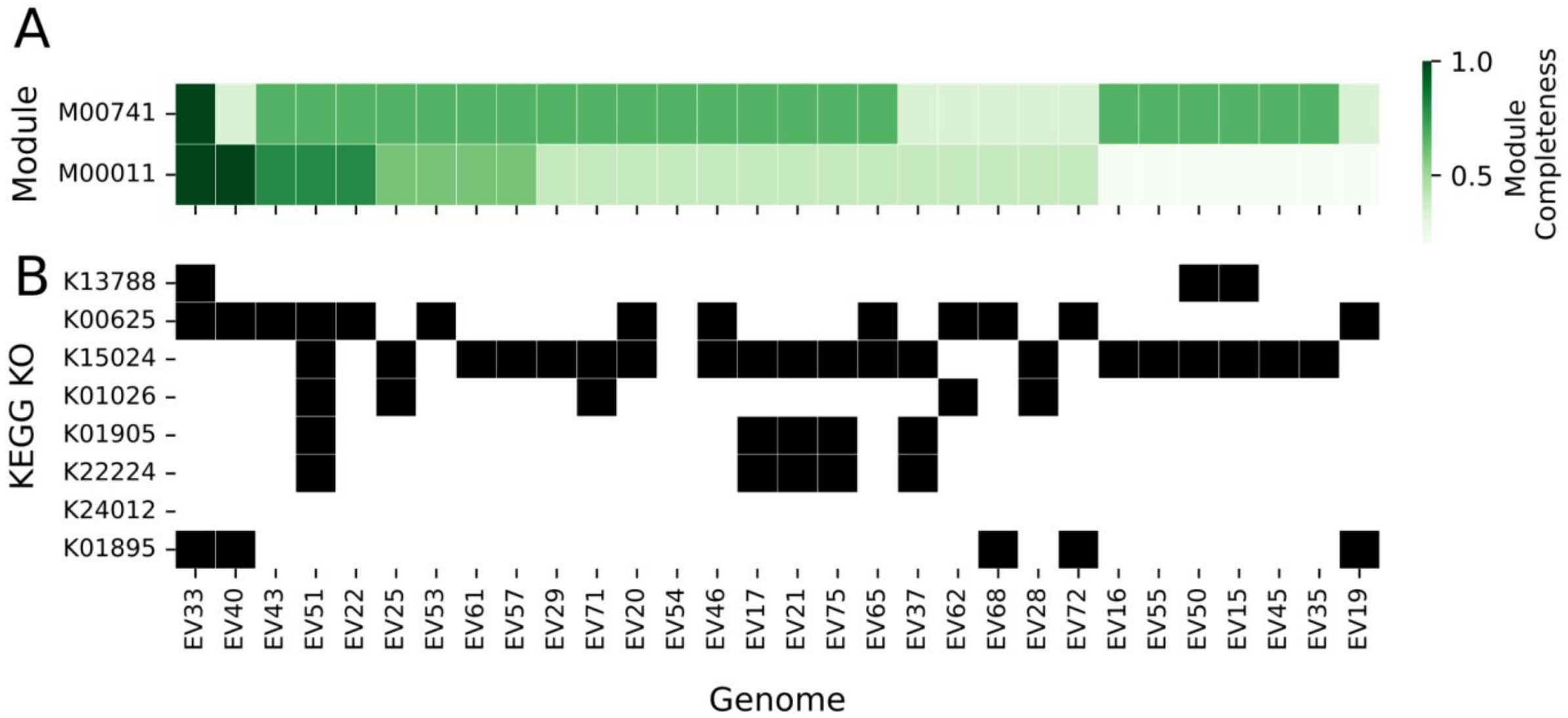

Fig. S4. Genomic potential of propionate production-related functions across MAGs. A) Heatmap showing the completeness of key KEGG metabolic modules, including M00741 (propanoyl-CoA metabolism) and M00011 (complete second carbon oxidation (citric acid cycle) across genomes; B) Presence–absence matrix of genes involved in the conversion of propionate to propionyl-CoA, representing the activation step required to channel propionate into downstream central metabolism (M00741) as defined on KEGG. This includes genes encoding phosphate acetyltransferase (K13788, K00625), phosphotransacetylase-like enzymes (K15024), propionate CoA-transferase (K01026), acdAB complex (K01905, K22224, K24012), and acetyl-CoA synthetase (K01895). Gene presence was inferred from annotated KO within each MAG. Black indicates presence.

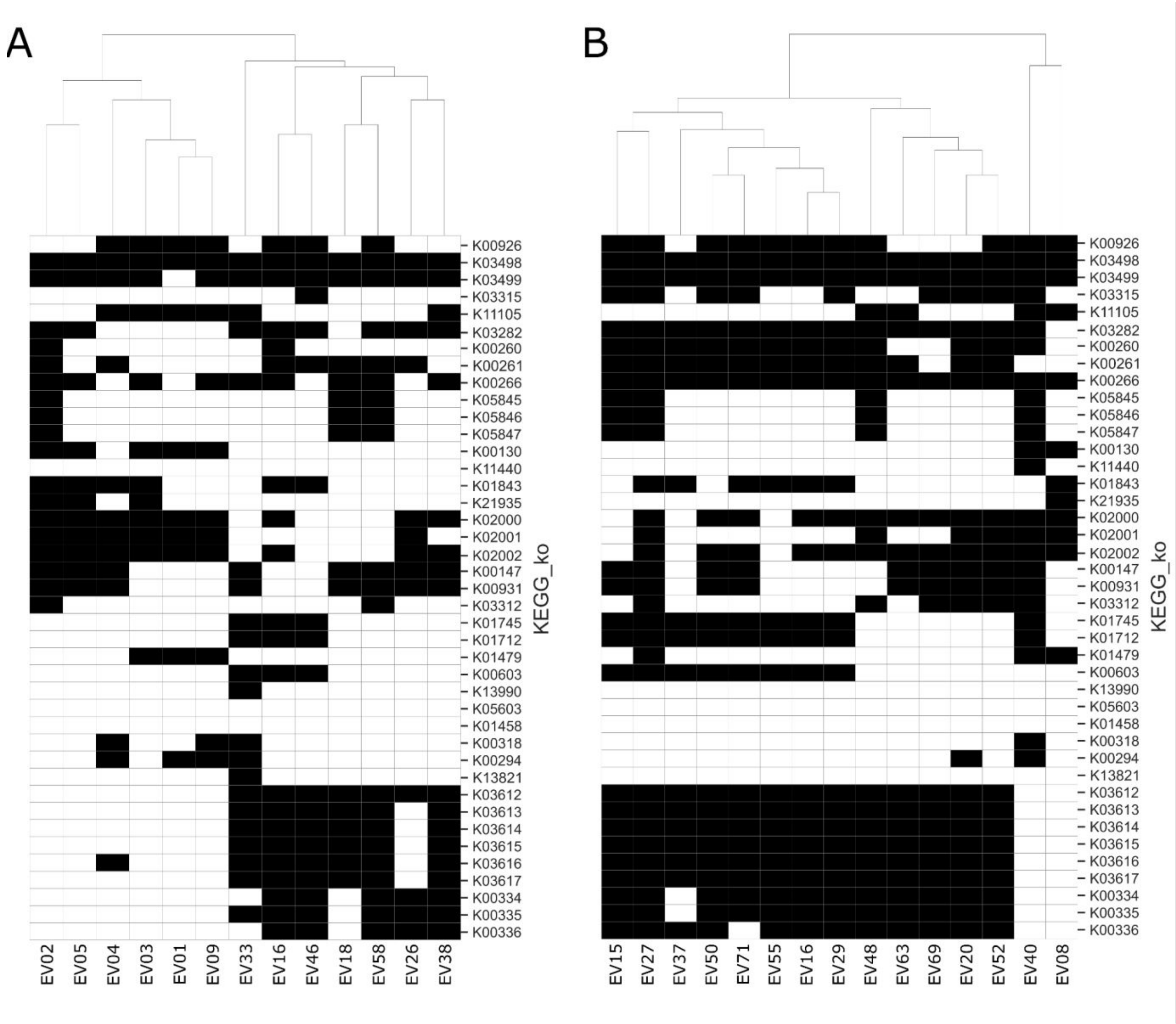

Fig. S5. Presence or absence of genes involved in NH_3 resistance, including osmoprotectant production and uptake, ion transport systems, and energy conserving complexes, comparing G1 (A) and G10 (B).

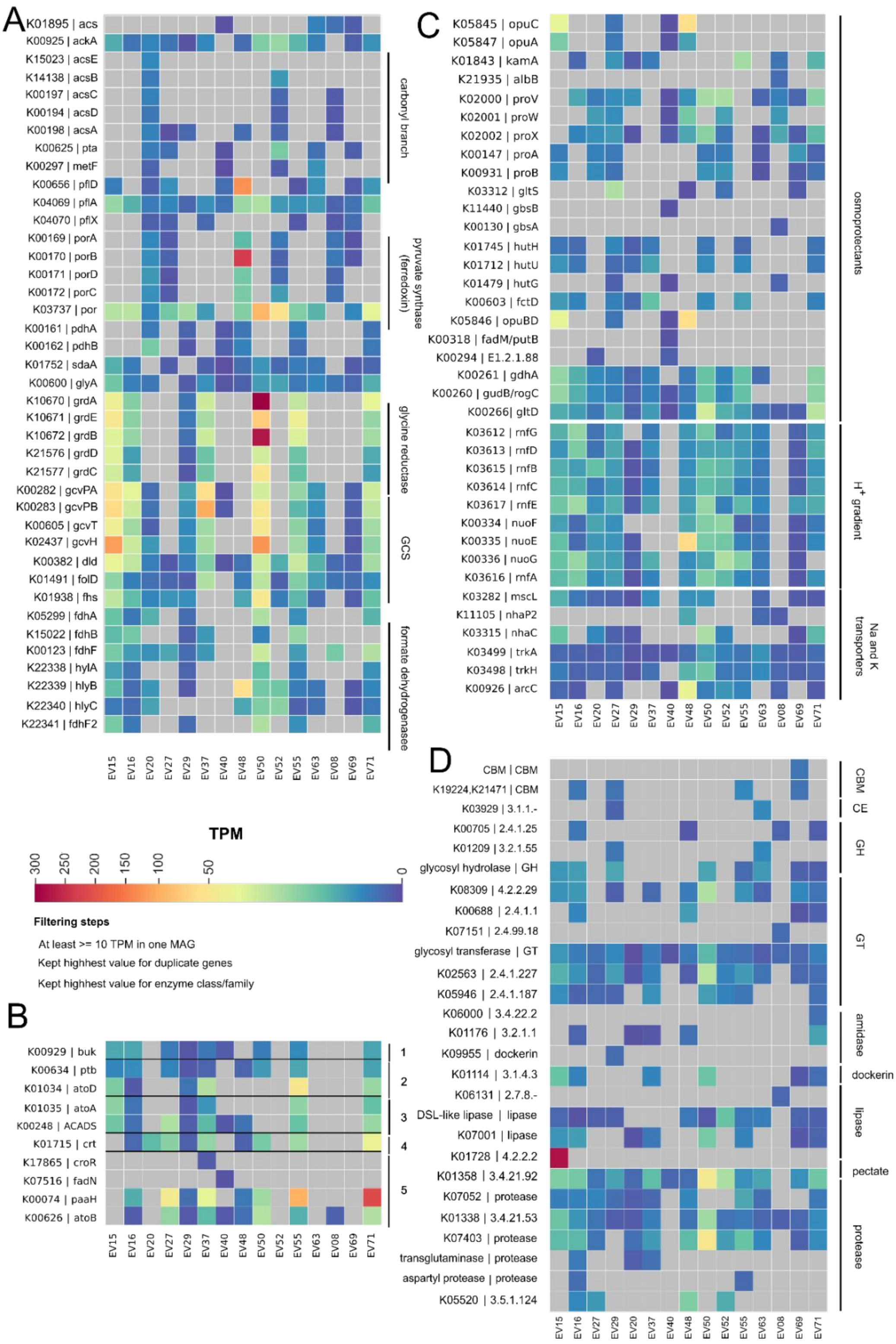

Fig. S6. DNA-normalized gene expression in the G10 community. For each gene, transcriptional activity is expressed as the transcript-to-genomic abundance ratio (TPM_RNA_/TPM_DNA_), reflecting expression relative to gene copy number.

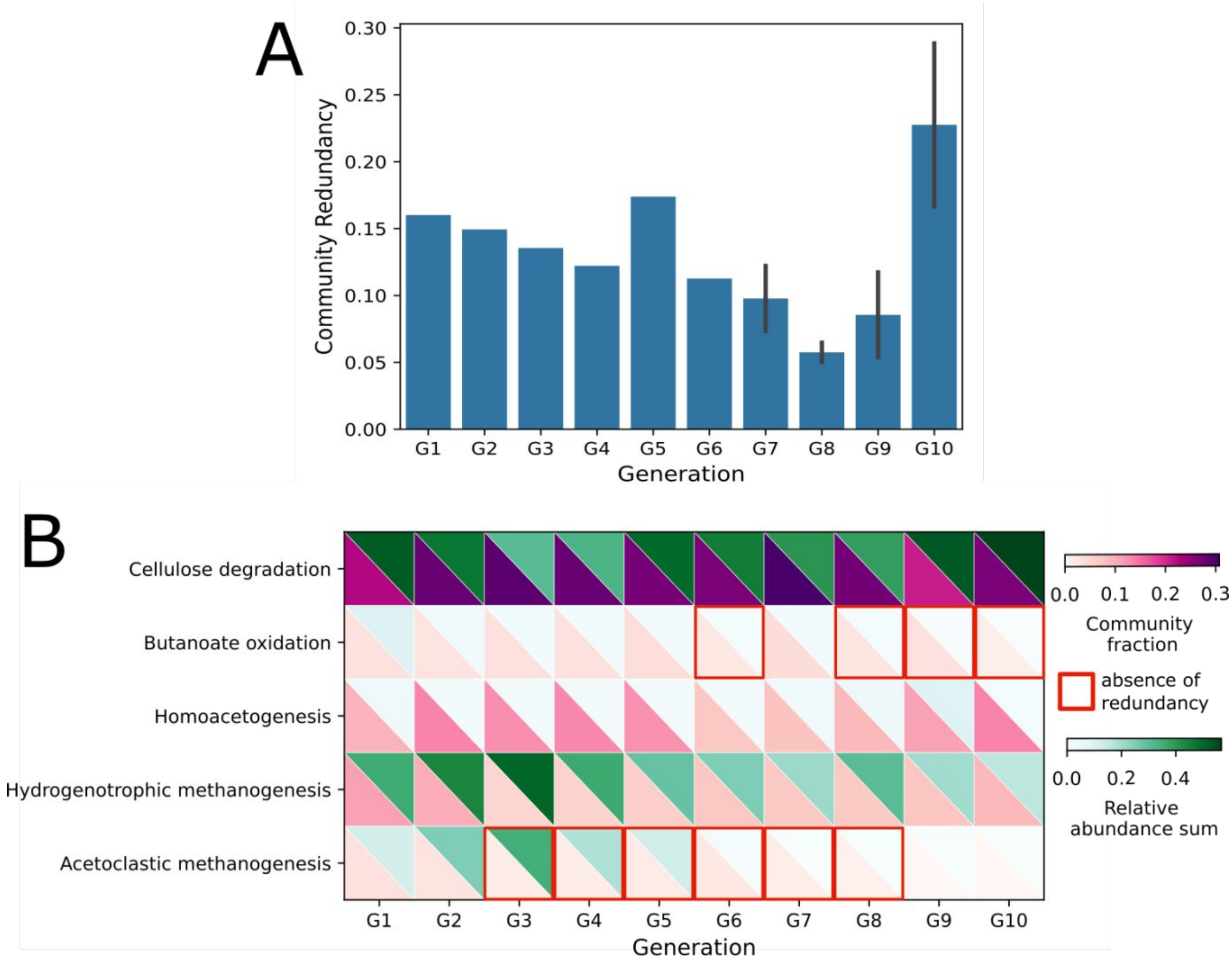

Fig. S7. Evolution of functional redundancy across experimental generations at both (A) community and (B) functional group levels. Redundancy progressively decreases from early stages to G9, followed by a marked increase in G10. Cellulose degradation and hydrogenotrophic methanogenesis consistently represent the most functionally redundant processes.
